# IFSEMC 2022: International Festival of Sports, Exercise & Medicine Conference: A joint conference between SASMA, LTMIC, PASHDA and SASB

**DOI:** 10.17159/2078-516X/2022/v34i1a14885

**Published:** 2022-01-01

## Background

Osteoarthritis (OA) is a major cause of disability, affecting an estimated 20% of the world population. Researchers have found that whilst individuals with unilateral lower limb amputation (ULLA) are five times less likely to develop OA in their prosthetic limb, they are 17 times more likely to develop OA in their sound side limb.

## Methodology

Researchers systematically searched PubMed-Medline, EBSCOhost and Web of Science for articles published between Jan 1990 and 28 February 2022. Studies were included if they investigated the involved side of individuals with OA and/or the uninvolved side of individuals with unilateral lower limb amputation. Whilst comparing the biomechanical variables (ground reaction force (GRF); external knee adduction moment (KAM); external knee adduction moment loading rate (KAM-LR); external knee adduction moment impulse (KAM-imp); knee flexion moment (KFM)) to either the contralateral side or a separate control group. Additionally, this study assessed the differences in the loading parameters between the involved side and the uninvolved side of these individuals.

## Results

Of the potential 496 eligible articles, 34 articles were included in this review. Twenty of the included studies investigated the involved side of an OA population and 14 studies the uninvolved side of an individual with ULLA. Variables, KAM and KFM, increased in both the individuals with ULLA and those with OA. For the GRF and KAM-LR, the OA population tended to show a decrease, while it increased for those with ULLA. For individuals with ULLA, KAM-imp had no known effect but increased in those with OA.

## Conclusion

While a vast amount of research exists on the development of OA in able-bodied individuals, few studies give a clear indication of the development of OA in individuals with ULLA. Studies often stated that individuals with ULLA are more likely to develop OA and present with abnormal ranges of the applicable variables, like in individuals with OA. Surrogate measures for contact force in the medial knee (KAM and KFM) exhibited the same trends in both population groups. Thus, a conclusion may be drawn that individuals with ULLA may demonstrate similar biomechanical profiles to individuals with diagnosed OA.

