# Supplementary material for: IFSEMC 2022: International Festival of Sports, Exercise & Medicine Conference: A joint conference between SASMA, LTMIC, PASHDA and SASB
Source: S Afr J Sports Med. 2022 Jan 1;34(1):v34i1a14885. doi: 10.17159/2078-516X/2022/v34i1a14885 (PMC9924557; doi:10.17159/2078-516X/2022/v34i1a14885)
Supplement: Related texts

# Abstract: O02: MANAGEMENT OF PROXIMAL RECTUS FEMORIS INJURIES – DO WE KNOW WHAT WE’RE DOING?: A SYSTEMATIC REVIEW

**Published:** 2022-01-01

**Authors:** Lone Bogwasi, Louis Holtzhausen, Dina C (Christa) Janse van Rensburg, Audrey Jansen van Rensburg, Tanita Botha

**Affiliations:** 1 Section Sports Medicine, Faculty of Health Sciences, University of Pretoria, Pretoria, South Africa; 2 CAF Medical committee member; 3 Botswana Football Association medical committee member; 4 Aspetar Orthopedic and Sports Medicine Hospital, Weill-Cornell Medical College; Qatar; 5 Department of Exercise and Sports Sciences, University of the Free State, Bloemfontein, South Africa; 6 Medical Board Member, World Netball, Manchester, UK; 7 Department of Statistics, Faculty of Natural and Agricultural Sciences, University of Pretoria, Pretoria, South Africa

## Background

Rectus femoris (RF) injury is a concern in sports. The management of RF strains/tears and avulsion injuries need to be clearly outlined. The purpose of this systematic review is to report the existing best evidence on current management strategies for RF injuries based on the current concepts in management of RF injuries. We ascertained the efficacy of these management strategies as measured by time to return to sport (RTS) and re-injury rates.

## Methodology

A systematic review of available literature was conducted using Medline via PubMed, WorldCat, EMBASE, SPORTDiscus. Eligible studies reporting on the management of RF kicking and sprinting injuries were reviewed.

## Results

Thirty-eight studies involving a hundred and fifty-two participants were included. Majority (n=138; 91%) were males, 80% (n=121) sustained RF injury from kicking and 20% (n=31) during sprinting. The myotendinous (MT), (n=27); free tendon (FT), (n=34), and anterior-inferior iliac spine (AIIS), (n=91) were involved. Treatment was conservative (n=115) or surgical (n=37) across the subgroups. The majority of surgical treatments (n=27; 73%) followed, failed conservative treatment. The mean RTS was shorter with successful conservative treatment (MT: 1, FT: 4, AIIS avulsion: 2.9 months). Surgical RTS ranged from 2–9 months and 18 months with labral involvement. With either group, there was no re-injury within 24 months of follow-up.

## Conclusion

With low certainty of evidence, RF injury occurs mostly from kicking, resulting in a tear or avulsion at the FT and AIIS regions with or without a labral tear. With low certainty, findings suggest that successful conservative treatment provides a shortened RTS. Surgical treatment remains an option for failed conservative treatment of RF injuries across all injury subgroups. High-level studies are recommended to improve the evidence base for the treatment of this significant injury.

# Abstract: O03: FOOT MORPHOLOGY OF URBAN AND RURAL SOUTH AFRICAN CHILDREN AND ADOLESCENTS

**Published:** 2022-01-01

**Authors:** Marise Breet, Ranel Venter

**Affiliations:** 1 Stellenbosch University

## Background

Ill-fitting shoes could negatively impact the development of the pediatric foot. This could eventually lead to foot problems and pathologies, both during childhood and adulthood. A substantial number of children and adolescents in South Africa were reported to be habitually barefoot. Additionally, vast differences have been documented between the foot morphology of South African children and adolescents and their German counterparts. Regardless of these findings, shoe companies are still developing shoes on a universal constant.

## Methodology

A cross-sectional, observational, descriptive study design was used to determine the static standing and sitting foot measurements of children and adolescents from both urban and rural schools. The feet of seven-hundred-and-thirty-one school children (N=731) between the ages of six and sixteen years were measured on a once-off basis. Newly manufactured school shoes were then measured and compared to the foot measurements of the children. A mixed model ANOVA was used to compare foot dimensions between gender, age and right- or left-side of the participants.

## Results

The results indicated that the rural children and adolescents had a statistically higher sitting static arch height index (p=0.001), than the urban children. There were no statistically significant differences in the standing foot length (FL), standing foot width (FW) and standing static arch height index (sAHI) between the feet of children and adolescents from rural and urban areas. Comparing the foot measurements of the participants in the study to the shoe dimensions currently available in the South African retail market, fifty-nine percent (59%) of the children wore shoes that were not the correct length. Regarding shoe width, ninety-eight percent (98%) of the participants wore shoes too narrow for their feet.

## Conclusion

Therefore in many cases, school shoes currently available in the retail market are not adequately suited for the habitually barefoot population studied. Factors such as footwear habits and ethnicity could be investigated further. It is recommended that the shoe manufacturing industry consider the shoe width of school shoes for South African children and adolescents, to avoid the long-term adverse effects of ill-fitting shoes on the pediatric foot, specifically in the case of habitually barefoot populations.

# Abstract: O04: AN ANALYSES OF TWITTER-TRAFFIC ON PHYSICAL ACTIVITY-RELATED MESSAGING DURING THE PERIOD OF SOUTH AFRICA’S COVID-19 LOCKDOWN

**Published:** 2022-01-01

**Authors:** Darren Cecil, Johan van Heerden

**Affiliations:** Dpt Biokinetics, UKZN, South Africa

## Background

Implementing a lockdown aided in controlling the COVID-19 pandemic but simultaneously hindered physical activity, leading to a health decline in many individuals regardless of activity status. Social media platforms such as Twitter were subsequently employed to communicate public health messages and to promote physical activity participation as a means to boost individuals’ immune systems. Accordingly, this study analysed the themes and trends of physical activity-related messaging on Twitter-Sphere, during the period corresponding with South Africa’s COVID-19 lockdown.

## Methodology

A content analysis using the PICO approach of conducting systematic reviews was utilized. The Tweets were collected from South Africa’s lockdown level 5 to level 1 (23 March 2020 to 5th November 2020) using 12 physical activity-related terms and/or hashtags (#) with 3 COVID-specific hashtags (#COVID, #COVID19 and #COVID_19). Sub-set categories of data were summarized as frequencies (n) and relative frequencies, with statistical significance between sets determined using the chi-square statistic with alpha set at p≤0.05.

## Results

A total of 1380 physical activity-related Tweets were analysed, with the greatest number posted during lockdown level 5 (n=385; 27.9%) with a decrease (p≤0.0001) thereafter as lockdown levels and their restrictions eased. The majority (p≤0.0001) of messages contained offering intents (96.4%) versus seeking intents (3.6%). Information was consistently the most offered subcomponent across all COVID lockdown levels. Tweets with seeking intents mostly sought responses to questions or surveys. Most Tweets (p≤0.0001) used text only (59,60%) as opposed to additional media in the form of images (29,5%), video (7,7%) and infographics (3.2%).

## Conclusion

Twitter has been a busy channel for the dissemination of physical activity-related information during the COVID-pandemic. The frequency of Tweets decreased over the duration of the pandemic. Most Tweets offered information in the form of simple text messages and images rather than seeking responses. This provides an understanding of the nature of physical activity-related tweeting over the COVID-19 lockdown period and insight in the face of potential future public health crises.

# Abstract: O05: PREVALENCE AND CORRELATES OF ADHERENCE TO TOTAL PHYSICAL ACTIVITY GUIDELINES USING STEP-COUNTING IN PRE-SCHOOL CHILDREN: THE SUNRISE STUDY

**Published:** 2022-01-01

**Authors:** Tawonga W. Mwase-Vuma, Xanne Janssen, Kar Hau Chong, Mark S. Tremblay, Amanda E. Staiano, Anthony D Okely, Catherine E. Draper, Nyaradzai Munambah, Penny Cross, John J. Reilly

**Affiliations:** 1 University of Strathclyde, Glasgow, UK; 2 University of Wollongong, Wollongong, New South Wales, Australia; 3 CHEO Research Institute, Ottawa, Canada; 4 Louisiana State University, Louisiana, US; 5 University of the Witwatersrand, Johannesburg, South Africa; 6 University of Zimbabwe, Harare, Zimbabwe

## Background

Evidence suggests that the De Creamer threshold of 11,500 steps/day is valid and internationally suitable for surveillance of the WHO recommendation that young children should spend at least 3 hours per day in total physical activity (TPA). However, there are limited studies on the prevalence and correlates of adherence to this guideline. Therefore, we examined the prevalence and identified correlates of adhering to the step-based TPA guideline using the De Craemer step count threshold of 11,500 steps/day in a geographically and culturally diverse sample of pre-school children.

## Methodology

This study included 797 pre-school children (49.2% girls, mean age 4.4 years) from 17 middle- and high-income countries who participated in the SUNRISE international pilot study. Steps/day were measured using activPAL accelerometers which children wore on the thigh for at least three days. Children were classified as meeting or not meeting the step-based TPA guideline of 11,500 steps/day. Logistic regression was used to identify potential socio-demographic correlates of meeting the step-based TPA guideline using the forward selection method. These included sex, age, parent/caregiver education class, residential area, and country income level.

## Results

Overall, 30.9% of the pre-schoolers met the step-based TPA threshold of at least 11,500 steps/day. The prevalence was higher among boys (34.3%) than girls (27.3%). The odds of meeting the step-based TPA threshold were significantly lower among girls (OR: 0.48; 95% CI: 0.31, 0.73) and 4-year-olds (OR: 0.36; 95% CI: 0.22, 0.58) vs. 3-year-olds. The odds of meeting the threshold were significantly higher among children from rural areas (OR: 2.28; 95% CI: 1.47, 3.54) vs. urban areas and lower-middle income countries (OR: 2.10; 95% CI: 1.27, 3.45) vs. high-income countries. Parent/caregiver education level was not associated with meeting the step-based TPA threshold in the adjusted model (OR: 2.19; 95% CI: 0.99, 2.47).

## Conclusion

Few pre-school children in this diverse global sample met the step count threshold that aligns with the WHO TPA guideline. Meeting the guideline was more prevalent among the younger children, boys, and children residing in rural areas and in lower-middle income countries.

# Abstract: O06: DISEASE BURDEN OF SOIL TRANSMITTED HELMINTH INFECTIONS ON CARDIOVASCULAR DISEASE RISK, PHYSICAL ACTIVITY AND FITNESS IN PRIMARY SCHOOL CHILDREN IN PORT ELIZABETH, SOUTH AFRICA

**Published:** 2022-01-01

**Authors:** Siphesihle Nqweniso, Cheryl Walter, Markus Gerber, Rosa du Randt, Johanna Beckmann, Jean Coulibaly, Kurt Z. Long, Harald Seelig, Jürg Utzinger, Christin Lang

**Affiliations:** 1 Department of Human Movement Science, Nelson Mandela University, Port Elizabeth, South Africa; 2 Department of Sport, Exercise and Health, University of Basel, Basel, Switzerland; 3 Swiss Tropical and Public Health Institute, Basel, Switzerland; 4 Centre Suisse de Recherches Scientifiques en Côte d’Ivoire, Abidjan, Côte d’Ivoire; 5 Unité de Formation et de Recherche Biosciences, Université Félix Houphouët-Boigny, Abidjan, Côte d’Ivoire

*(Abstract withdrawn)*

# Abstract: O07: THE EPIDEMIOLOGY OF LOWER LIMB INJURIES DURING THE SUPER RUGBY TOURNAMENTS (2013–2016) INVOLVING 76301 PLAYER-HOURS

**Published:** 2022-01-01

**Authors:** Clement Plaatjies, Audrey Jansen van Rensburg, Martin Schwellnus, Christa Janse van Rensburg, Charl Janse van Rensburg, Esme Jordaan

**Affiliations:** 1 Section Sports Medicine, Faculty of Health Sciences, University of Pretoria, Pretoria, South Africa; 2 Sport Exercise Medicine and Lifestyle Institute (SEMLI), Faculty of Health Sciences, University of Pretoria, Pretoria, South Africa; 3 Emeritus Professor of Sport and Exercise Medicine, Faculty of Health Sciences, University of Cape Town, South Africa; 4 International Olympic Committee (IOC) Research Centre, South Africa; 5 Biostatistics Unit, South African Medical Research Council, South Africa; 6 Statistics and Population Studies Department, University of the Western Cape

## Background

Rugby union involves frequent high-impact collisions and tackles and has a high injury rate. The aim was to determine the incidence, nature (player position, anatomical body area, tissue type), severity (% time loss, injury burden), mechanism of injury (contact vs. non-contact), and phase of play of lower limb injuries (LLI) sustained by rugby players during the Super Rugby tournaments.

## Methodology

A cross-sectional analysis studied 868 male professional rugby union players from all South African teams participating in the Super Rugby tournament (2013–2016). Team physicians recorded all LLI (all training and match LLI) daily. Injury exposure was 76301 total player-hours, including 6520 match player-hours. Numbers, proportions, incidence (I: per 1000 player-hours; 95% CI), and LLI burden (days lost due to LLI per 1000 player-hours) are reported.

## Results

Throughout the 4-years, a total count of 776 injuries occurred. LLI attributed to 422 (54.4%) of all training and match injuries with an overall incidence of 5.3 (4.9–5.8). LLImatch injuries (LLImatch, n=346) contributed 51.2% to the total match injuries (n=676) with an incidence of 53.1 (47.6–59.0). The incidence of LLImatch injuries in backline players (52.6; 45.5–60.4) compared to forwards (46.9; 40.7–53.9) were similar (p>0.05). In match-play, the thigh (27.5%) and knee (24.0%) were the most commonly affected anatomical body area. Muscle/tendon injuries comprised 57.8% of all LLImatch injuries, and joint/ligament injuries of 35.3%. Most match injuries occurred in contact (61.0%), and during a tackle situation (42.2%). The majority of LLImatch injuries were of minimal to mild severity (2–7 days; 60%). The injury burden of LLImatch injuries was 1126 (1100–1152) days lost.

## Conclusion

LLI are the most common injury sustained by male Super Rugby players, especially during match-play (51.2%). The thigh and knee are the most frequent lower limb area injured. Most LLI during matches are muscle and tendon injuries and are of minimal to moderate severity. The most significant contributor to LLImatch injury is contact and the tackle situation. These findings are important for designing and implementing LLI prevention strategies in rugby players.

# Abstract: O08: PHYSICAL ACTIVITY, FITNESS AND VISUAL ATTENTION IN YOUNG ADULTS WITH DEPRESSIVE SYMPTOMS

**Published:** 2022-01-01

**Authors:** Sarah J Moss, L Enslin, A Kruger, S.H. Czyz

**Affiliations:** 1 PhASRec, North-West University, Potchefstroom; 2 CHHP, North-West University, Potchefstroom; 3 PhASRec, North-West University, Potchefstroom and Faculty of Physical Education and Sport, University School of Physical Education, Wrocław, Poland

## Background

The management of mental health in young South African adults is a health priority. Like the global trends, mental health, including Major Depression, is increasing among young adults. Inverse associations are reported between depression scores and screen time, but the association with visual attention, as determined with multiple object tracking (MOT) task, have not been investigated. This study aims to determine the associations between depressive symptom score, physical activity, fitness level, screen time and MOT in young South African adults with depressive symptoms.

## Methodology

Baseline preliminary data from 30 young adults aged 18 – 25 years enrolled in the Depression exercise intervention study were analysed. The Beck Depression Inventory (BDI) was used to screen for depressive symptoms among the participants. Physical measurements of body composition, objective physical activity (ActiHeart) and fitness were assessed. Cortisol was measured from blood samples and visual attention with MOT (Neuro Tracker). Associations between the variables were determined with Pearson correlation analyses.

## Results

Participants (aged 21 ± 2 years) had an average BDI-score of 19.8 ± 9.1 and an average body fat percentage of 30 ± 12%. Less than 80 minutes per week was spent in moderate-to-vigorous physical activity, and the predicted fitness was 35.1 ± 6.5 ml/kg/min. MOT response time was 8.15 ± 1.29 sec. Significant correlations were found between the BDI score and Leisure screentime (r = 0.512; p =0,005), as well as BDI score and MOT total time (r= −0.529; p = 0.007).

## Conclusion

This study concludes that persons reporting high screen time during leisure also scored higher for depressive symptoms. Higher depressive symptoms scores were correlated with lower visual attention total time. Although a causal effect cannot be determined, understanding the relationships between screen time, visual attention, fitness and depressive symptoms will assist in developing intervention strategies for the management of depressive symptoms in young adults.

# Abstract: O09: MENTAL HEALTH PROFILES OF HEALTHCARE PROFESSIONALS WORKING DURING THE TOKYO 2020 AND BEIJING 2022 PARALYMPIC GAMES

**Published:** 2022-01-01

**Authors:** Maaike Eken, Phoebe Runciman, Marelise Badenhorst, Leslie Swartz, Vincent Gouttebarge, Cheri Blauwet, Wayne Derman

**Affiliations:** 1 Institute of Sport and Exercise Medicine (ISEM), Department of Sport Science, Faculty of Medicine and Health Sciences, Stellenbosch University, Cape Town, South Africa; 2 Ports Performance Research Institute New Zealand (SPRINZ), Auckland University of Technology, Auckland, New Zealand; 3 Department of Psychology, Stellenbosch University, Stellenbosch, South Africa; 4 Department of Orthopaedic Surgery, Amsterdam Movement Sciences, Amsterdam UMC, University of Amsterdam, Amsterdam, Netherlands; 5 Section Sports Medicine, University of Pretoria, Pretoria, South Africa; 6 Department of Physical Medicine and Rehabilitation, Spaulding Rehabilitation Hospital and Brigham and Women’s Hospital, Harvard Medical School, Boston, Massachusetts, USA; 7 International Olympic Committee Research Centre, South Africa

## Background

During the Paralympic Games, healthcare professionals (HCPs) play an important role in managing athletes’ health. HCPs are however required to work long hours, and stress and exhaustion are not uncommon during Games time. These factors could contribute to adverse mental health of HCPs who treat Para athletes during Games, which could influence the (mental) health of the athletes. The aim of this study is to describe mental health profiles of HCPs in the Paralympic setting.

## Methodology

HCPs working during the Tokyo 2020 and Beijing 2022 Paralympic Games were asked to complete an online, anonymous survey, which included demographic questions and questions regarding self-reported mental health symptoms including depression (PHQ-9), anxiety (GAD-7) and burn-out (MBI/HSS: depersonalisation (DP), emotional exhaustion (EE), personal accomplishment (PA)). Frequency analyses were used to describe demographic characteristics and mental health symptoms. Spearman’s rho was calculated to investigate correlations between demographic characteristics and mental health symptom scores.

## Results

In total, 256 HCPs (of approximately 500 HCPs) completed the surveys, of which 212 were from Tokyo and 44 Beijing. The majority of HCPs practiced medicine (56%), were more than 10 years involved in management of athlete health (52%) and from a high-income economy country (46%). Most HCPs reported minimal (72%) or mild (16%) depressive symptoms and minimal (72%) or mild anxiety related symptoms (19%), while more than 30% reported moderate to high burnout (DP: 35%; EE: 35%; PA: 56%). Yet, severe symptoms and thoughts of self-harm were reported by some HCPs. Weak correlations were observed between age and depression (rho=−0.13, p=0.046), anxiety (rho=−0.16, p=0.010) and aspects of burnout (EE: rho=−0.14, p=0.032; PA: rho=0.27, p<0.001) scores, indicating that symptoms of depression, anxiety and burnout were more reported by younger HCPs.

## Conclusion

Most HCPs working during the Tokyo 2020 and Beijing 2022 Paralympic Games reported minor mental health symptoms, yet some concerning reports were noted. Findings suggested also that symptoms of depression, anxiety and burnout could be more prevalent among younger HCPs. Teams that are travelling to Paralympic Games (or other major tournaments) are therefore advised to provide guidance and mental health support of young(er) HCPs travelling with the team.

# Abstract: O10: AFRICAN BIG-5 SPORT PSYCHOLOGICAL ASSESSMENT TOOL: DEVELOPMENT AND PRELIMINARY VALIDATION

**Published:** 2022-01-01

**Authors:** Heinrich Grobbelaar

**Affiliations:** 1 Division of Sport Science, Department of Sport Science, Faculty of Medicine and Health Sciences, Stellenbosch University

## Background

The assessment of psychological skills, techniques and methods through valid and reliable self-report instruments are important for sport psychology consultants, both from a theoretical and applied perspective. The study aimed to determine the psychometric properties of Wheaton’s (1998) Psychological Skills Inventory (PSI). Due to poor Confirmatory Factor Analysis (CFA) results on 1610 data entries, the follow-up aim was to develop and validate a new tool to assess sport psychological skills and mental states of athletes.

## Methodology

The dataset was randomly split-in-half, yielding a train and test set with 805 data entries each. An Exploratory Factor Analysis (EFA) on the train data suggested a five-factor assessment tool. Items that loaded (> 0.40) onto the five subscales were screened. Two CFAs followed on the train and test sets respectively, to test the goodness-of-fit of the 40-item instrument.

## Results

Both CFAs demonstrated acceptable goodness-of-fit, item and construct reliability, standardised estimates, and extracted variance results. The five subscales were matched to the African Big-5 animal that represented it best: Freedom from competition worries (Buffalo), Goal setting (Leopard), Visualisation (Elephant), Competition confidence (Lion), and Competition concentration (Rhino).

## Conclusion

The study demonstrated preliminary evidence in support of the African Big-5 Sport Psychological Assessment Tool’s (Big-5 SPAT) validity and reliability. Future studies should cross-validate the instrument, determine its discriminant and convergent validity and usefulness in applied settings, translate it into other indigenous African languages, and develop normative data.

# Abstract: O11: CORRELATIONS BETWEEN FREQUENCY OF PARTICIPATION IN CAMPUS RECREATION, LEISURE BOREDOM, AND SATISFACTION WITH LIFE OF UNDERGRADUATE STUDENTS

**Published:** 2022-01-01

**Authors:** Theron Weilbach, Nadine Labuschagne, Theron Weilbach, Cornelia Schreck

**Affiliations:** 1 PhASRec, NWU

## Background

Emerging adulthood is a challenging life stage and distress, loneliness, and even disengagement in their academic career can hinder students’ transition from high school to university, and consequently affect their entire university experience. However, campus recreation (CR) can play a significant role in the transition from school to university. The purpose of this study was to determine the correlation between the frequency of participation in CR, leisure boredom, and satisfaction with the life of undergraduate students of the North-West University.

## Methodology

A once-off, cross-sectional research design, with a quantitative approach was used. In total, 581 students (48% males; 52% females) completed an online survey which included demographic information, frequency of participation, the format in which each activity is presented, the satisfaction with life scale by Diener et al. (1985), and leisure boredom items from the Leisure Experience Battery for Young Adults by Barnett, (2005).

## Results

Activities participated in were grouped into six different categories, namely main university sports, additional university sports, group sports, outdoor activities, dance, and exercise. Spearman correlation coefficients were used to determine the relationships between different variables. In terms of relationships between leisure boredom and satisfaction with life, a negative statistically significant correlation (r=−.170, p=0.000) exists. Total participation in CR had a significant positive correlation with satisfaction with life (r=0.135, p=0.001), and a significant negative correlation with leisure boredom (r=−0.146, p=0.000). In terms of correlations between participation in specific categories of CR, satisfaction with life, and leisure boredom, the following was revealed: statistical significant correlations exist between satisfaction with life and additional sport codes (r=.0.90, p=0.029), outdoor sports (r=0.143, p=0.001), and exercise sport (r=0.99; p=0.017). Additionally, statistically significant negative correlations were found between leisure boredom and additional sport codes (r=−0.095, p=0.022), group sports (r=−.0.089, p=0.032), dance (r=−0.085, p=0.040), outdoor sports (r=−.149, p=0.000) and exercise sports (r=−0.099, p=0.017).

## Conclusion

The findings show the importance of CR during students’ time at university. It also gives insight to recreation professionals on which programme areas may benefit students the most.

# Abstract: O12: PREVALENCE OF THE RELATIVE AGE EFFECT AMONG HIGH PERFORMANCE, UNIVERSITY ATHLETES, VERSUS AN AGE-MATCHED STUDENT COHORT

**Published:** 2022-01-01

**Authors:** Sindiso Dube, Heinrich Grobbelaar

**Affiliations:** 1 Division of Sport Science, Department of Sport Science, Faculty of Medicine and Health Sciences, Stellenbosch University

## Background

Relative age effect (RAE) refers to the over-representation of athletes born earlier in the calendar year. The RAE is especially prevalent in youth sport, but often persists into senior competitive levels. The aim was to determine the prevalence and magnitude of the RAE among student-athletes in a high-performance (HP) programme at a South African university, according to year, sport code and sex, compared, to the general student cohort.

## Design

Cross-sectional descriptive analysis of HP-student-athletes and an age-matched student cohort from 2016 to 2021.

## Methods

Birthdate data was extracted for the HP student-athletes (N = 950: men = 644, women = 306) and student comparison group (N = 47 068; men = 20 464; women = 26 591; not disclosed = 13). Differences were determined using Chi-squared and Fisher’s exact test. Residuals examined relative age quartile differences. The steps were applied across academic years, sport code and sex.

## Results

The RAE was more pronounced among the student-athletes compared to the age-matched student cohort and seems to become more apparent with each passing year. When analysing the different sport codes, RAEs were observed for swimming, rugby union and cricket. No sex differences were observed among the HP-student-athletes.

## Conclusions

Selection bias favoured the relatively older student-athletes. The mechanisms for RAE are multifactorial and complex. A combination of factors, such as competition depth, the popularity and physicality of a sport and socialization may be involved.

# Abstract: O13: IMPACT OF BIOLOGICAL MATURITY STATUS ON TALENTED AND NON-TALENTED MALE ZIMBABWEAN UNDER-14 FOOTBALLERS

**Published:** 2022-01-01

**Authors:** Joseph Syakhuluma Sibindi, Heather Morris-Eyton, Charl Roux

**Affiliations:** 1 University of Johannesburg

## Background

The study aimed to examine the impact of biological maturity status on identified talented and non-talented male Zimbabwean Under-14 footballers. Currently, the associated differences and impact of biological maturity status on talent identification and selection of youth footballers are unclear.

## Methodology

A total of 141 Under-14 footballers were purposively sampled from the Bulawayo Metropolitan and Matabeleland North provinces. The observational and field test methods were used. The non-hierarchical K-means cluster analysis classified 87 footballers as talented and 54 as non-talented using technical football skills tests.

## Results

The mean scores for the talented group were as follows: chronological age (CA) (12.075 ± 0.838), age at peak height velocity (APHV) (14.591 ± 0.558) and maturity offset (−2.614 ± 0.532) while the reported mean scores for the non-talented group were CA (11.914 ± 0.945), APHV (14.597 ± 0.283) and maturity offset (−2.614 ± 0.565). Footballers were further classified as early maturers (16.7%), normal maturers (67.9%) and late maturers 15.5% for the talented group. The non-talented group had 21.1% (early), 59.6% (normal) and 19.3% late maturers. Hochberg or Dunnett’s test indicated no differences among the early, normal and late maturers for the talented and non-talented groups (p > 0.05). There were significant differences between early and late maturers for selected anthropometric data and the shuttle dribble sprint test for the talented group (p < 0.05). In the non-talented group, the early, normal and late maturers showed significant differences for percentage body fat from the triceps and similarly for the late maturers compared with both the early and normal for the distance covered in the Yo-yo test. The late maturers in both groups had the fastest shuttle dribble sprint times and lower percentage body fat from the triceps skinfold.

## Conclusion

A normal distribution was observed for the talented and non-talented groups among the early maturers, normal maturers and late maturers. Overall, the differences among variables suggested that the maturity status had little or no influence in the talent identification and selection of Under-14 footballers. However, practitioners need to identify and select youth footballers based on their potential.

# Abstract: O14: PATELLOFEMORAL PAIN SYNDROME (PFPS) IS ASSOCIATED WITH CHRONIC DISEASE AND ALLERGIES IN 60 997 DISTANCE RUNNER RACE ENTRANTS: A SAFER STUDY

**Published:** 2022-01-01

**Authors:** Tsungai Marandure, Martin P. Schwellnus, Catharina Grant, Audrey Jansen van Rensburg, Esmè Jordaan, Pieter Boer

**Affiliations:** 1 Section Sports Medicine, Faculty of Health Sciences, University of Pretoria, South Africa; 2 Sport, Exercise Medicine and Lifestyle Institute (SEMLI) Pretoria, South Africa; 3 Biostatistics Unit, South African Medical Research Council, South Africa; 4 Statistics and Population Studies Department, University of the Western Cape, South Africa; 5 Department of Human Movement Science, Cape Peninsula University of Technology, South Africa

## Background

Patellofemoral Pain Syndrome (PFPS) is a common gradual onset running-related injury with multiple risk factors. This descriptive cross-sectional study aims to identify the risk factors associated with PFPS in distance runners that entered the 21.1km and 56km Two Oceans Marathon races (2012–2015).

## Methodology

A pre-race medical screening questionnaire was completed by 106 743 race entrants, 76 654 participants (71.8%) consented to the study. The study population (n=60 997) consisted of 362 race entrants reporting a history of PFPS in the previous 12 months, and 60 635 runners with no injury history. PFPS was verified by a health care professional in the injured group. Risk factors associated with PFPS were explored using uni- & multiple regression analyses: demographics (age groups, sex, and race distance), training/running variables, history of existing chronic diseases (including a composite chronic disease score) and any allergy history. Prevalence (%, 95%CI) and prevalence ratios (PR) are reported.

## Results

The period (12 month) prevalence of PFPS was 0.5% (0.43–0.54). Independent risk factors associated with a history of PFPS (adjusted for age, sex and race distance) were a higher chronic disease composite score (PR=2.7 times increased risk for every 2 additional chronic diseases; p<0.0001), and a history of any allergies (PR=2.3; p<0.0001). Chronic diseases (PR>2; univariate analysis) associated with a history of PFPS were: gastrointestinal disease (PR=5.1; p<0.0001), risk factor for cardiovascular disease (CVD) (PR=3.3; p<0.0001), nervous system/psychiatric disease (PR=3.0; p<0.0001), cancer (PR=2.8; p=0.0005), risk factors for CVD (PR=2.4; p<0.0001), symptoms of CVD (PR=2.4; p=0.0397) and respiratory disease (PR=2.0; p<0.0001). An increased number of years of recreational running (PR=1.1; p=0.0107) and older age (>50 years) were also associated with a higher risk of PFPS.

## Conclusion

The novel independent risk factors associated with a history of PFPS in distance runners (21.1km, 56km) are multiple chronic diseases and a history of any allergies. A medical evaluation to identify runners with chronic diseases may be considered a specific injury prevention strategy to reduce the risk of PFPS.

# Abstract: O15: AN ABBREVIATED VS. A COMPREHENSIVE PRE-RACE MEDICAL SCREENING TOOL UNDER-ESTIMATES RUNNERS AT HIGHER RISK OF MEDICAL ENCOUNTERS – A SAFER STUDY IN 5771 RACE ENTRANTS

**Published:** 2022-01-01

**Authors:** Jordan Leppan, Martin Schwellnus, Nicola Sewry, Jeremy Boulter, Christa Janse van Rensburg, Marlise Dyer, Esme Jordaan

**Affiliations:** 1 Section Sports Medicine, Faculty of Health Sciences, University of Pretoria; 2 Sport, Exercise Medicine and Lifestyle Institute (SEMLI), University of Pretoria; 3 IOC Research Centre, Pretoria, South Africa; 4 Comrades Marathon, Pietermaritzburg; 5 Biostatistics Unit, South African Medical Research Council (SAMRC); 6 Statistics and Population Studies, University of the Western Cape

## Background

The Comrades Marathon has, for many years, obtained medical information from participants through an abbreviated pre-race screening tool consisting of two open-ended medical questions. It is unknown how this abbreviated screening compares to full pre-race medical screening tools. The aim of the study was to determine if two pre-race screening tools (abbreviated tool of two open-ended pre-race medical screening questions [ABBR] vs. a full pre-race medical screening tool [FULL]) identify the same number of running race entrants at higher risk for medical encounters (MEs)-based on the identification of: 1) allergies 2) medical conditions and/or prescription medication use.

## Methodology

Data from 5771 consenting race entrants who completed both the ABBR and the FULL pre-race screening questionnaires for the 2018 Comrades Marathon were collected. The two ABBR questions were 1) allergies, and 2) known medical conditions and/or prescription medication use in free text. The FULL tool included multiple domains of questions for various diseases (including cardiovascular disease (CVD) symptoms and risk factors), running injuries, allergies and medication use. Responses to the ABBR were manually coded in similar domains of questions as in the FULL and compared to the FULL, and further risk stratified into “very high-risk”, “high risk”, intermediate risk” and “low risk” of having a medical encounter during the race (based on pre-existing medical conditions). The prevalence (%: 95%CI), and the test for equality of prevalence (with p-value) of entrants identified by the ABBR vs. FULL are reported.

## Results

The ABBR identified fewer entrants with allergies (ABBR=7.9%; FULL=10.4%: p=0.0001) and medical conditions/medication use (ABBR=8.9%; FULL=27.4%: p=0.0001). The FULL stratified many more entrants in the “high risk” (12.4%) and “very high-risk” (3.4%) categories compared with the ABBR (3.36% and 0.5% respectively) (p=0.0001). The ABBR also over-estimated the number of entrants in the “low risk” category (63.4%) compared to the FULL (46.5%).

## Conclusion

The ABBR significantly under-reported for allergies, specific medical conditions/medication use and risk profile of the entrants compared with the FULL. This level of under-reporting will lead to a false prediction of MEs on race day.

# Abstract: O16: DEVELOPMENT OF A TRAIL RUNNING INJURY SCREENING INSTRUMENT: A MULTIPLE METHODS APPROACH

**Published:** 2022-01-01

**Authors:** Carel Viljoen, Christa Janse van Rensburg, Willem van Mechelen, Evert Verhagen, Elzette Korkie, Tanita Botha

**Affiliations:** 1 Department of Physiotherapy, Faculty of Health Sciences, University of Pretoria, Pretoria, South Africa; 2 Amsterdam Collaboration for Health and Safety in Sports, Department of Public and Occupational Health, Amsterdam Movement Sciences, Amsterdam University Medical Centers, location VU University Medical Center, Amsterdam, The Netherlands; 3 Sport, Exercise Medicine and Lifestyle Institute (SEMLI), Pretoria, South Africa; 4 Section Sports Medicine, Faculty of Health Sciences, University of Pretoria, Pretoria, South Africa; 5 School of Human Movement and Nutrition Sciences, Faculty of Health and Behavioural Sciences, University of Queensland, Brisbane, Australia; 6 Division of Exercise Science and Sports Medicine (ESSM), Department of Human Biology, Faculty of Health Sciences, University of Cape Town, South Africa; 7 School of Public Health, Physiotherapy and Population Sciences, University College Dublin, Dublin, Ireland; 8 Department of Physiotherapy, Faculty of Health Sciences, University of Pretoria, Pretoria, South Africa; 9 Department of Statistics, Faculty of Natural and Agricultural Sciences, University of Pretoria, Pretoria, South Africa

## Background

Trail running has a high risk for injury and medical support is challenging in remote regions. Injury risk management in trail running is of importance, but limited literature is available in the field. Therefore, the need to make use of expert opinion to guide current injury risk management in trail running.

## Methodology

The study utilised five phases in a multiple methods approach 1) identification of injury risk factors 2) determining the relevance of each identified risk factor in a trail running context, 3) creating the content of the Likert scale points from 0 to 4, with each point indicating an increase in injury risk for each selected injury risk factor, 4) rescaling the Likert scale points to determine numerical values for the content of each Likert scale point, and 5) determining a weighted score for each injury risk factor that contributes to the overall combined composite score.

## Results

Seventy-seven injury risk factors were identified of which 26 risk factors were deemed relevant in trail running. The weighted score for each injury risk factor ranged from 2.21 to 5.53. The highest calculated scores were 5.53 (buying running shoes based on a running analysis and not primarily based on a good shoe fit), followed by 5.41 (not adhering to a specific running-related, supervised training plan), and 5.11 (competitive training). The final TRISI includes risk categories of training, running equipment, demographics, previous injury, behavioural, psychological, nutrition, chronic disease, physiological, and biomechanical factors

## Conclusion

The developed TRISI aims to assist the clinician during pre-race injury screening or during a training season to identify meaningful areas to target in designing injury risk management strategies and/or continuous health education

# Abstract: O17: FIVE INTERNATIONAL PRE-EXERCISE MEDICAL SCREENING TOOLS VARY GREATLY REGARDING THEIR IDENTIFICATION OF RUNNING RACE ENTRANTS WHO REQUIRE MEDICAL CLEARANCE: A SAFER STUDY

**Published:** 2022-01-01

**Authors:** Chanel Smith, Kim Nolte, Martin Schwellnus, Nicola Sewry, Sonja Swanevelder, Esme Jordaan

**Affiliations:** 1 Sport, Exercise Medicine and Lifestyle Institute (SEMLI) and Division of Biokinetics and Sports Science, Department of Physiology, Faculty of Health Sciences, University of Pretoria, South Africa; 2 International Olympic Committee (IOC) Research Centre, South Africa; 3 Emeritus Professor, Faculty of Health Sciences, University of Cape Town, South Africa; 4 Biostatistics Unit, South African Medical Research Council; 5 Statistics and Population Studies Department, University of the Western Cape

## Background

The purpose of pre-exercise medical screening is to identify individuals who may be at risk of medical encounters (MEs) during exercise. Currently five international pre-exercise medical screening tools are recommended to identify individuals who require pre-exercise medical clearance. The aim of this study was to determine the percentage of race entrants who are advised to obtain pre-exercise medical clearance by using five international pre-exercise medical screening tools, and to determine the level of agreement between those tools.

## Methodology

Data from running race entrants (Two Oceans Marathon) were collected over a period of four years (2012–2015). Five pre-exercise medical screening tools (the American Heart Association (AHA), the pre-2015 American College of Sports Medicine (ACSM), the post-2015 ACSM, the Canadian Physical Activity Readiness Questionnaire (PAR-Q), and the European Association of Cardiovascular Prevention and Rehabilitation (EACPR)) were applied by using information from pre-race medical screening questionnaires. The primary measure of outcome was the % (95%CI) of race entrants for whom pre-exercise medical clearance was recommended using each of the five international pre-exercise medical screening tools. A secondary measure was the level of agreement (Kappa test) between the results obtained by using different tools.

## Results

The percentage entrants requiring medical clearance for each tool was as follows: 2011 EACPR (33.9%; 33.5–34.3); pre-2015 ACSM (33.9%; 33.5–34.3); PAR-Q (23.2%; 22.9–23.6); AHA (10.0%; 9.7–10.2); post-2015 ACSM (6.7%; 6.5–6.9). The level of agreement was high between the pre-2015 ACSM and the EACPR (K=1.00; p=0.05), moderate between the pre-2015 ACSM and the PAR-Q (K=0.75; p<0.0001) and the PAR-Q and EACPR (K=0.75; p<0.0001), but poor between the post-2015 ACSM and the PAR-Q (K=0.17; p<0.0001).

## Conclusion

Five international pre-exercise medical screening tools vary greatly regarding their identification of race entrants identified requiring medical clearance (6.7–33.9%). The level of agreement between the tools also varied and was good (> 0.75) for three of the five pre-exercise medical screening tools. Further research should determine which specific variables identify participants at higher risk for medical encounters during exercise.

# Abstract: O18: THE PREVALENCE OF MENTAL HEALTH DISORDERS AMONG SEMI-PROFESSIONAL CRICKETERS RESIDING IN THE WESTERN CAPE

**Published:** 2022-01-01

**Authors:** Lesego Malele, Habib Noorbhai

**Affiliations:** 1 Department of Sport and Movement Studies, Faculty of Health Sciences, University of Johannesburg, South Africa

## Background

Cricket requires high cognitive function for optimum performance. Due to COVID-19, it is even more paramount that mental health of athletes is monitored. The existing epidemiology studies among cricket players are equivocal; and as a result, the prevalence and long-term effects of mental health disorders is unknown. The aim of this research study was to investigate the prevalence of mental health disorders among semi-professional cricketers (provincial B and university squads) and how it affects their well-being.

## Methodology

This was an epidemiological, cross-sectional study design. The study was conducted among semi-professional (provincial B and university squads) cricket players (n = 63) residing in the Western Cape. Among the sample, descriptive statistics including means and standard deviations were calculated. Mental health screening among cricketers was assessed with The Depression, Anxiety and Stress Scale – 21, (DASS – 21), The Satisfaction with Life Scale (SWLS) and Athlete Burnout Questionnaire.

## Results

The DASS – 21 subscales are depression (M = 9.90, SD = 9.36), which translated mild depression. Anxiety (M = 10.67, SD = 9.60) was translated to moderate anxiety and stress (M = 14.73, SD = 9.85) means that most players fell within the mild stress category. The SWLS (M = 27.71, SD = 6.61) means that the players are slightly satisfied; but there are areas requiring improvement. The athlete burnout subscales are physical and emotional exhaustion (rarely; M = 23.2, SD = 3.90); devaluation of sport practice (almost never; M = 29.2, SD = 6.61) and reduced sense of achievement (sometimes; M = 23.4, SD = 2.97).

## Conclusion

The results depicts that some players require mental health investigation, as it is paramount for their performance. Burnout, mental health as well as life satisfaction plays a vital role in an athlete being successful. Cricket South Africa would improve holistically if mental health monitoring of cricketers are integrated in their existing systems, which can indirectly provide better performance management of players. Since there is limited evidence on the psychological effects on performance among cricketers at an amateur and professional level, future studies should widen investigations of mental health disorders among varied skilled levels.

# Abstract: O19: THE RELATIONSHIP BETWEEN TACKLE TECHNIQUE MEASURED IN TRAINING AND MATCHES

**Published:** 2022-01-01

**Authors:** Steve den Hollander, Mike Lambert, Ben Jones, Sharief Hendricks

**Affiliations:** 1 Research Centre for Health through Physical Activity, Lifestyle and Sport (HPALS), Dept of Human Biology, Faculty of Health Sciences, University of Cape Town; 2 UXI Sport; 3 CARR; 4 Leeds Beckett University

## Background

Contact (tackle, ball-carry, and ruck) technique is associated with measures of performance and injury outcomes in rugby matches and training. Yet, little is known about the relationship between contact technique assessed in training and matches, or the transference of a player’s technique proficiency in training into matches. This study aimed to describe the relationship between contact technique in training and matches, and measures of performance.

## Methodology

Twenty-four male players, from an amateur rugby club, participated in the study. At the beginning of the season, players’ contact technique proficiency was assessed in a training drill. Contact technique in matches was assessed during 14 competitive league matches. The technique proficiency was assessed using standardised criteria, and the outcomes of each tackle, ball-carry and ruck were recorded. A one-way analysis of variance, Cohen’s effect sizes and linear regression analyses were used.

## Results

Higher contact technique scores were associated with positive performance outcomes in training and in matches. For instance, the tackle technique was significantly lower in missed, compared to ineffective, tackles in training (p<0.05, ES=0.8) and matches (p<0.05, ES=1.2). Players’ contact technique scores in matches also had a positive effect on their match performance. Ball-carry technique was associated with tackle breaks made per game (p<0.05, r2 = 0.31). However, contact technique scores in training did not affect match performance. Contact technique scores were also lower in matches compared to training, with a 13.3% difference in means between ruck technique assessed in training and matches (p<0.01, ES=0.8). We found a large variation in the percentage of a player’s training technique represented in their match technique, with tackle technique ranging from 68%–100%.

## Conclusion

The findings of this study demonstrate the validity of assessing contact technique in training, as technique proficiency led to the same outcomes in the tackle, ball-carry, and ruck, in training and matches. The differences in technique between training and matches indicate that the drill may be too structured to fully represent the open nature of the tackle and ruck contests. These findings highlight the importance of contact skill training, in environments of varying structures, to ensure skills developed in training are transferred to match performance.

# Abstract: O20: SOCCER PLAYERS’ PERCEPTION OF SOCCER BOOT COMFORT - A STUDY TO INVESTIGATE SOCCER BOOTS’ SUITABILITY TO SOCCER PLAYERS’ FOOT MORPHOLOGY

**Published:** 2022-01-01

**Authors:** Simiso Ntuli, Heather Morris-Eyton, Andrew Green

**Affiliations:** 1 University of Johannesburg

## Background

The foot is a complex structure used for locomotion, which has great malleability and susceptibility to internal and external factors. It must be rigid to carry bodyweight with appropriate stability and must interact and adapt to all ground conditions; therefore, it must be flexible to support the body during static and dynamic tasks. A soccer boot is the most crucial piece of equipment for soccer players that has two primary functions, performance enhancement and injury prevention. Additionally, it acts as an interface between the player’s foot and the playing surface as well as between the player’s foot and the ball. Foot morphology and boot match should be considered significant in the provision of soccer injury prevention strategies. However, few studies have investigated the soccer boot’s suitability to the foot type of the intended user. The aim of this study was to investigate soccer players’ foot morphology and the suitability of soccer boots.

## Methodology

A cross-sectional design study in which data were collected from professional and university soccer players. Data were collected in two phases. Phase one included the completion of an adapted questionnaire to collect data on player demographic data including areas of pain or discomfort with the boot. Phase two involved scanning each participant’s feet using a 3D scanner to determine each player’s foot morphology and measurements. Data were analysed using descriptive statistics with a P-value set at < 0.05 for statistical significance.

## Results

Data were collected from professional (n=127) and university (n=62) male soccer players. The mean age was 25.09 and 20.21years respectively, with mean playing years reported at 6.75 and 2.71. Most players (92%) reported areas of discomfort and reduced fitting with their current boot. Players identified the toe box (94%), arch area (77%) and boot length (18%) as areas of pain or irritation. The 3D measurements revealed variations in arch heights, forefoot width and hallux toe flexion angles.

## Conclusion

There is evidence that players experience discomfort with their current soccer boots. This could be due to a disjuncture between foot type and boot fit. Foot morphology is an important consideration when selecting a soccer boot to match the individual foot type. Considering foot morphology during boot selection will improve the boots’ two primary functions, performance enhancement and injury prevention

# Abstract: O21: INDEX OF COORDINATION OF RUNNING IN TRIATHLETES

**Published:** 2022-01-01

**Authors:** Katelyne Van Den Bergh, Ryan Raffan

**Affiliations:** 1 Nelson Mandela Univeristy

## Background

Motor organization in swimming has been measured using the Index of Coordination (IdC). However, the IdC has not been utilized to analyse other cyclic actions such as running. This study aimed at investigating the IdC of running in triathletes.

## Methodology

Sixteen male triathletes participated in two consecutive, self-paced 5000m shuttle run trials, with full recovery between trials. The trials were video recorded but only the fastest trial was coded frame-by-frame to determine the time markers of heel strike and toe-off for both legs, and used to calculate IdC. The IdC was calculated for each of the three portions of the 5000m trial (1650m, 1650m and 1700m). Backward stepwise multiple regression was performed. The group was dichotomized into a fast and slow running group using the median split technique and a t-test was used to determine significance between groups.

## Results

The results revealed that 48% of the fastest trial run time can be explained by the IdC obtained in portion three (p=0.003). Furthermore, the slower running group had a significantly lower IdC in portion one (p=0.033) whereas the faster running group had a significantly lower IdC in portion three (p=0.008).

## Conclusion

These findings suggest that the movement system of the faster running group was able to produce better adaptive responses as they progressed through the 5000m, demonstrating a more efficient and economical organizational mode. In contrast, the movement system of the slower running group struggled to adapt to the task demands as they progressed through the 5000m, particularly in the later part of the task, resulting in significant increases in IdC and a more inefficient mode of coordination. The IdC should be utilized as a performance tool, in conjunction with other performance parameters, to help identify motor organization and inefficiencies. Lastly, investigating the influence of motor synchronization devices on the emergence of more adaptive and efficient motor responses may be worthwhile.

# Abstract: O22: THE EFFECT OF TIBIALIS ANTERIOR WEAKNESS ON FOOT DROP AND TOE CLEARANCE IN PATIENTS WITH FACIOSCAPULOHUMERAL DYSTROPHY

**Published:** 2022-01-01

**Authors:** Clement N. Gambelli, Jonathan Bredin, Aude-Clemence M. Doix, Jeremy Garcia, Veronique Tanant, Manuella Fournier-Mehouas, Claude Desnuelle, Sabrina Sacconi, Serge S. Colson

**Affiliations:** 1 Physical activity, Sport and Recreation Research Focus Area (PhASRec), Potchefstroom Campus, North-West University (NWU), Potchefstroom, South Africa; 2 Université Côte d’Azur, LAMHESS, France; 3 Laboratory of Physiology and Biomechanics of Locomotion, Institute of Neuroscience (IoNS), Universite catholique de Louvain (UCL), Louvain-la-Neuve, Belgium; 4 Centre de Santé Institut Rossetti-PEP06, Nice, France; 5 Université Côte d’Azur, CHU, France; 6 Université Côte d’Azur, CNRS, Inserm, iBV, France; 7 Université Côte d’Azur, CHU, CNRS, Inserm, IRCAN, France

## Background

Facioscapulohumeral dystrophy (FSHD) is a genetic disease characterized by progressive muscle weakness leading to a complex combination of postural instability, foot drop during swing and compensatory strategies during gait that have been related to an increased risk of fall. The aim is to assess the effect of Tibialis Anterior muscle (TA) weakness on foot drop and minimum toe clearance (MTC), as well as the compensatory strategies of FSHD patients during gait.

## Methodology

Eight FSHD patients allocated to a subgroup depending on their TA weakness assessed by manual muscle testing (i.e., FSHD<3 and FSHD>3) and 8 matched healthy control participants were equipped with retro-reflective markers to record the kinematics of walking at self-selected speed with a motion analysis system.

## Results

Walking speed (for both FSHD<3 and FSHD>3) and step length (for FSHD<3 only) were significantly decreased in FSHD patients whereas MTC central tendency was similar across all FSHD patients and control participants. A greater foot drop was systematically observed for FSHD<3 during swing and only in late swing for FSHD>3.. In addition, MTC variability was increased only for FSHD<3. Individual strategies to compensate for foot drop remains unclear and may depend on other muscle impairment variability.

## Conclusion

The reduced walking speed for all FSHD patients seems to be a compensatory strategy to maintain balance although trunk instability and foot drop at foot strike. The more precautious gait in patients with severe TA weakness, i.e. reduced step length, seems to be an adaptation to enhanced foot drop. Increased MTC variability in FSHD patients with severe TA weakness suggests an increased risk of tripping. Manual muscle testing is a simple and effective method to assess TA weakness and seems to identify FSHD patients at an increased risk of tripping.

# Abstract: O23: THE ACUTE EFFECTS OF SCHOOLBAG LOAD ON STATIC AND DYNAMIC KINETICS AND KINEMATICS IN 10–13-YEAR-OLD CHILDREN

**Published:** 2022-01-01

**Authors:** Mark Kramer, Bridget Grobler

**Affiliations:** 1 North-West University

## Background

Excessive schoolbag loading may lead to unfavourable postural deviations and musculoskeletal pain. Curvatures of the spine can be influenced by asymmetrical loading of the schoolbag, or by muscle imbalances between the dominant and non-dominant sides of the body, leading to (i) compensatory shifts in static and dynamic body postures, (ii) adjusted postural sway, and (iii) deviations in gait.

## Methodology

Sixty participants volunteered for the study and were subcategorised by age: 10 year-olds (n = 15), 11 year-olds (n = 15), 12 year-olds (n = 16), and 13 year-olds (n = 14). Participants were evaluated for changes in (i) craniovertebral and sagittal shoulder postures, (ii) postural sway, and (iii) gait kinetics and kinematics during loaded and unloaded conditions.

## Results

Approximately 58% of participants exceeded a schoolbag mass of 15% BM, and 27% exceeded a schoolbag mass of 20% BM. Significant differences were evident for those reporting pain and exceeding the 15% BM threshold compared to those with pain but below the 15% BM threshold (χ2 (1) = 5.79, p = 0.016, relative risk [RR] = 2.63, 95% CI [1.04, 6.62]). Deviations were evident between loaded and unloaded conditions for: (i) craniovertebral angles (Mdiff = −1.70 deg, 95% CI [−3.25, −0.15]) and sagittal shoulder angles (Mdiff = 3.08 deg, 95% CI [−0.03, 6.19]), (ii) postural sway (Mdiff = 70.29 mm2, 95% CI [−63.55, 204.14]), and (iii) all plantar force (Mdiff = 14–40 N) and pressure (Mdiff = 44–199 N/cm2) values, as well as gait velocity (Mdiff = −0.17 km.hr−1, 95% CI [−0.29, −0.04]); although none of these reached practical significance (Cohen’s d: −0.28–0.26).

## Conclusion

Schoolbag loads significantly influenced plantar pressures and forces as well as sagittal shoulder postures, but not postural sway or gait velocity. Most of the individuals evaluated exceeded the 15% BM threshold, and those exceeding the 15% BM threshold experienced 2.63 times the risk of experiencing pain compared to those below the threshold. Therefore, schoolbag loads should be kept below 15% of BM.

# Abstract: O24: CLUSTERED CARDIOVASCULAR DISEASE RISK AMONG 8 TO 13-YEAR-OLD CHILDREN FROM LOWER SOCIOECONOMIC SCHOOLS IN GQEBERHA, SOUTH AFRICA

**Published:** 2022-01-01

**Authors:** D Dolley, C Walter, R du Randt, U Pühse, J1 Bosma, J Degen, N Joubert, P Arnaiz, I Müller, M Gerber

**Affiliations:** 1 Nelson Mandela University; 2 University of Basel; 3 Swiss Tropical and Public Health Institute

## Background

It is estimated that 51% of South African deaths can be accounted for by non-communicable diseases (NCDs) yet just over half of South African children comply with the recommendation of 60 minutes of moderate to vigorous physical activity (MVPA) per day. The aim of the study was to determine the prevalence of individual and clustered cardiovascular disease (CVD) risk factors among children from lower socio-economic communities and to investigate the independent association between clustered CVD risk, MVPA, and cardiorespiratory fitness (CRF).

## Methodology

Baseline data derived from the KaziBantu study were collected in a cross-sectional analysis of 975 children, aged 8–13-years-old from eight quintile 3 schools. Measurements included height, weight, waist circumference, blood pressure, fasting glucose, full lipid panel, 20 m shuttle run performance and accelerometery. The prevalence of individual CVD risk factors was determined using standardised cut-offs, and a clustered risk score (CRS) was constructed using principal component analysis. Participants with an elevated CRS of 1SD above the average CRS were considered “at-risk”.

## Results

We found 424 children (43.3%) having at least one elevated CVD risk factor: 27.7% elevated triglycerides, 20.7% depressed HDL-C and 15.9% elevated total cholesterol. An elevated clustered risk was identified in 17% (n=104) of the sample; girls exhibited a significantly higher CRS>1SD than boys (p=0.036). The estimated odds of an elevated clustered risk are doubled every 2 mL/kg/min decrease in VO2max (95% CI: 1.60–2.40 mL/kg/min) or every 50 min reduction in MVPA (95% CI: 1.27–4.33 min).

## Conclusion

A relatively high prevalence of elevated individual and clustered CVD risk was identified. Our results have also confirmed the independent inverse association of the clustered CVD risk with PA and CRF, respectively, and indicate that increased levels of CRF or MVPA may aid in the prevention and reduction of elevated clustered CVD risk. These findings emphasise the importance of an active lifestyle to counteract early-life CVD risk in under-served communities and schools such as those in the Gqeberha, Eastern Cape region of South Africa.

# Abstract: O25: DOES PHYSICAL ACTIVITY, IN PART, EXPLAIN THE LOWER-THAN-EXPECTED MORTALITY FROM COVID-19 IN SUB-SAHARAN AFRICA?

**Published:** 2022-01-01

**Authors:** Estelle Victoria Lambert

**Affiliations:** 1 Research Centre for Health through Physical Activity, Lifestyle and Sport (HPALS), Dept of Human Biology, Faculty of Health Sciences, University of Cape Town

## Background

The recent pandemic has highlighted the link between chronic, non-communicable diseases (NCDs) and associated risk factors, that predict poor prognoses and severity of outcomes in relation to Covid-19. Globally, the infectious disease burden is typically inequitably distributed, with LMICs experiencing the greatest share. However, in a recent publication by the New York Times, journalists posited that Sub-Saharan Africa had experienced a lower-than-expected number of “excess deaths” due to Covid-19. The potential explanations offered included: younger age distribution, low testing rates, rural and undocumented deaths, and a more physically active population, spending more time outdoors. The latter explanation was discussed in a recently published commentary in Progress in Cardiovascular Diseases (Wachira et al., in press, https://doi.org/10.1016/j.pcad.2022.04.012). The aim of this preliminary investigation was to explore publicly-available data, in order to provide further insights into this phenomenon.

## Methodology

Publicly available data for 136 countries (17=LIC, 35=LMIC, 39=UMIC, 45=HIC) were gathered from the WHO Global Health Observatory, the World Bank and Worldometer (aggregated data in real-time concerning population, government, economics, environment, energy and health). Variables included: Total population, tests per million, cases per million, deaths per million, GINI coefficient, World Bank classification, percentage urbanised, and physical inactivity prevalence. Linear regression analyses were conducted to predict COVID-19 outcomes.

## Results

Descriptive data for countries by World Bank classification showed that urbanisation ranged from 37.2% in LICs to 75.6% in HICs (P<0.001). Similarly, physical inactivity prevalence was lowest in LICs at 18% and highest in HICs at 33.6% (P<0.001). Covid-19 cases/deaths per million ranged from 3119 and 53, respectively in LICs to 255516 and 1816, respectively, in HICs (P<0.001). Linear regression models including % inactivity (P=0.008), % urban (P=0.012) and HIC vs others (P=0.025) explained 27% of the variance in deaths per million. A similar model, substituting Sub-Saharan Africa for HICs, explained a similar % of the variance in deaths per million (R2=0.274) and was protective. Physical inactivity was not implicated in cases per million.

## Conclusion

Preliminary analyses suggest that per capita Covid-19 mortality is linked to physical inactivity, urbanisation and is greatest in high-income countries. Covid-19 cases were not linked to physical inactivity in this sample. These results provide indirect support for the role of physical activity in mitigating Covid-19 severity, although more research is needed.

# Abstract: O26: BRING IN THE BIOKINETICISTS: FACILITATING BEHAVIOUR CHANGE IN A LOW-RESOURCED COMMUNITY

**Published:** 2022-01-01

**Authors:** Sweetness J Makamu-Beteck, Francois G Watson, Melainie Cameron, Sarah J Moss

**Affiliations:** 1 PhASRec, North-West University, Potchefstroom; 2 Numiq, North-West University, Potchefstroom; 3 School of Health and Medical Sciences, University of Southern Queensland, Autralia

## Background

To interpret and discuss the contributions of supervised exercise compared to standard clinic care on the perceptions and knowledge of risk factors for non-communicable diseases (NCDs) and physical activity (PA) among women living in a low-resource setting in South Africa.

## Methodology

From 200 recruited participants, 172 women were assigned to either 24 weeks of supervised exercise training (intervention group), n=95 or 24 weeks of standard clinic care (control group), n=77. We used a convergent parallel mixed method to collect qualitative data and quantitative data at baseline, 12-weeks, and 24 weeks of the intervention. Qualitative explorations consisted of focus group discussions assessing perceptions of NCDs and PA. Quantitative measurements included knowledge surveys of heart disease, biological (blood pressure, peripheral blood glucose and total cholesterol), body composition (weight, height, waist, and hip circumference) measures, and objective PA measurements over seven consecutive days of free-living with combined heart rate van accelerometry (ActiHeart^®^). Qualitative content analysis was done using Atlas.Ti. Quantitative data were analysed using linear mixed models. The Health Belief Model was applied as the lens through which the mixed methods data were described and interpreted.

## Results

The participants’ perceptions and understanding of NCD risk factors improved more among the supervised exercise group than in the control group. Both groups reported significant increases from baseline to 24 weeks in PA knowledge (p ≤ 0.001) and heart disease knowledge (p ≤ 0.001). Significant improvements in DBP (p ≤ 0.001), SBP (p≤0.001) and waist circumference (p=0.03) were reported for both groups. The exercise group showed a significant faster rate of reduction as compared with the control group in SBP (β = 4.10, t = 1.97, p = 0.05) and DBP (β = 3.81, t = 2.97, p ≤ 0.001). The control group had faster rate of reduction in waist circumference (β = −2.46, t = −2.68, p = 0.01).

## Conclusion

The supervised exercise intervention improved perceptions of risk factors for NCD and PA after 24-weeks when participating in a supervised exercise intervention compared to women receiving standard clinic care.

Trial registration: PACTR201609001771813

# Abstract: O27: ENERGY EXPENDITURE, INTENSITY, AND PERCEIVED EFFORT IN RECREATIONAL FUNCTIONAL TRAINING

**Published:** 2022-01-01

**Authors:** Lucilia Mangona, Ledda Almeida Brasil, Antonio M. Prista, Paulo T. Farinatti

**Affiliations:** 1 Higher School of Sports Science, Maputo, Mozambique; 2 Graduate Program in Exercise and Sport Sciences, University of Rio de Janeiro State, Rio de Janeiro, Brazil; 3 Research Group for Physical Activity and Health, Faculty of Physical Education and Sports, Pedagogical University of Mozambique, Maputo, Mozambique

## Background

Few studies have estimated the energy expenditure (EE) of functional training (FT) sessions, none of which are in actual conditions in training centres or involving recreational practitioners. This information would be useful to better understand the potential of TF as a strategy to achieve adequate physical activity volumes to promote health. The objective of this Doctoral Thesis was to quantify the EE during FT sessions using triaxial accelerometry, in adults without previous experience with the modality, in a commercial gym in the city of Maputo (Mozambique). The relative and absolute intensity and rate of perceived effort (RPE) were also assessed. Additionally, data from FT were compared with those derived from continuous walking sessions (WLK).

## Methodology

Twenty-five volunteers with no previous experience with FT (11 men, 16 overweight, 38.8 ±9.3 years; 73.9±13.8 kg; 168.5 ± 8.5 cm) performed three training sessions interspersed with 48h (two familiarization and one assessment). FT circuit included four rounds of 12 exercises performed at all-out repetitions during 20 s, with 10-s intervals between rounds. WLK was performed for 25 min with intensity corresponding to scores 3–5 at Borg CR-10 Scale.

## Results

Outcomes were EE (kcal) and movement counts estimated by a triaxial accelerometer worn at the waist; heart rate reserve (%HRR); and RPE. FT sessions lasted on average 24 min and EE ranged between 124–292 kcal (188±41 kcal), corresponding to 5–8 METs (6.1±0.6 METs), and 70–80 %HRR (74±8%). The rate of movements (counts/min) showed that vigorous predominated over moderate intensity during FT and WLK (p=0.01), with similar EE. However, the relative intensity (74% vs. 55 %HRR, respectively; p=0.0001) and RPE (Borg 5–8 vs. 3–5, respectively; p=0.0001) were higher in FT vs. WLK.

## Conclusion

In conclusion, a short recreational FT circuit applied at a conventional fitness centre elicited EE and intensity compatible with recommendations to reduce cardiometabolic risk and improve cardiorespiratory fitness in participants with normal or overweight. This training modality should be considered as an alternative for health-oriented exercise programs for the general population.

# Abstract: O28: ENVIRONMENTAL FACTORS INCREASE THE RISK OF HEAT/ELECTROLYTE DISORDERS IN ULTRA-MARATHON RACES

**Published:** 2022-01-01

**Authors:** Marlise Dyer, Esme Jordaan, Henno Havenga, Nicola Sewry, Martin Schwellnus

**Affiliations:** 1 Sport, Exercise Medicine and Lifestyle Institute (SEMLI), University of Pretoria; 2 Biostatistics Unit, South African Medical Research Council (SAMRC), Department of Statistics and Population Studies, University of the Western Cape; 3 Department of Environmental Sciences and Management, North-West University; 4 Sport, Exercise Medicine and Lifestyle Institute (SEMLI), University of Pretoria, IOC Research Centre, Pretoria, South Africa

## Background

The Comrades Ultra Marathon over 90km takes place every year between the towns of Pietermaritzburg and Durban. The event alternates between up runs (Durban to Pietermaritzburg) and down runs (Pietermaritzburg to Durban), over an undulating course. Participants typically start the race between 5h29 and 6h04, depending on the individual seeding positions. Participants are differentially exposed to various weather conditions along the route, most notably heat stress exposure which has been shown to contribute to medical-related illnesses during the race. The aim was to explore the ability of two indices, the Wet-bulb globe temperature (WBGT) and the Universal Thermal Comfort Index (UTCI), to predict the risk of athletes developing heat/fluid conditions (heat illness, rhabdomyolysis, Fluid and electrolyte disorders which included hypothermia, hyperthermia/exertion heat stroke, dehydration, (mild, moderate or severe), hyponatremia, other electrolyte disorders).

## Methodology

Two indices, namely the Wet-bulb globe temperature (WBGT) and the Universal Thermal Comfort Index (UTCI), are explored to derive the environmental conditions on race day. Daily weather conditions for the period 2014–2019 were gathered from South-African Weather Service (SAWS) weather stations in proximity of the race route along with ERA5 Reanalysis Data that represents a modelled environment of past weather conditions for the region. The WBGT and UTCI values were derived from these data sources for every hour from 05h00 until 18h00 and a cumulative heat exposure value was derived for each athlete. Heat/electrolyte data from the down races for the years 2014, 2016, and 2018 were analysed.

## Results

The correlation between the WBGT and UTCI values was 0.82. In total 443 athletes were treated for heat/electrolyte disorders. The prevalence of electrolyte disorders was 1.1% (1.0 to 1.2%). The modelling of heat/fluid conditions with UTCI showed a significant increase in risk with an increase in UTCI (PR=1.23 for every 2 units increase in UTCI; 95%CI: 1.16–1.3), p=0.0001) and with WBGT showed a significant increase in risk with an increase in WBGT (PR=1.7 for every 2 units increase in WBGT; 95%CI: 1.4–1.9), p=0.0001)

## Conclusion

In both WBGT and UTCI, an increase indicates an increased risk of heat/electrolyte disorders in ultra-marathon races.

# Abstract: O29: THE RISK MARKERS ASSOCIATED WITH ACUTE RESPIRATORY TRACT ILLNESS IN RUGBY PLAYERS DURING THE SUPER RUGBY TOURNAMENTS (2013–2017), INVOLVING 102 738 PLAYER-DAYS

**Published:** 2022-01-01

**Authors:** Audrey Jansen van Rensburg, Dina C. (Christa) Janse van Rensburg, Martin P. Schwellnus, Charl Janse Van Rensburg, Esmé Jordaan

**Affiliations:** 1 Section Sports Medicine, Faculty of Health Sciences, University of Pretoria, Pretoria, South Africa; 2 Sport, Exercise Medicine and Lifestyle Institute (SEMLI), Faculty of Health Sciences, University of Pretoria, Pretoria, South Africa; 3 Biostatistics Unit, South African Medical Research Council, South Africa

## Background

Acute respiratory tract illness (aRTill) presents a significant health burden to elite rugby players and can disrupt training and competition performance. Data of possible risk markers for aRTill in rugby players are limited. The aim was to determine the associated risk markers of aRTill in rugby players during the Super Rugby tournaments.

## Methodology

Team physicians completed daily illness logs in 537 professional male rugby players from all South African teams participating in the 2013–2017 Super Rugby tournaments (exposure: 1141 player-seasons, 102,738 player-days). Information recorded includes player factors [age (years), height (cm); weight (kg); player position (forwards, backs), illness previous year], team factors [different teams], season factors [competitive season (year), phase of season (early, mid, late), number of matches (≤8, >8)], travel factors [home/away, last 7 days], location, match days and maximum wet bulb globe temperature (WBGT). Main outcome measures included numbers, proportions, incidence (I: per 1000 player-days; 95% CI) and incidence ratio (IR: per 1000 player-days: 95%CI).

## Results

Throughout the 5-years, 305 aRTill were reported with an overall incidence of 2.9 (2.6–3.3). In the univariable regression analysis, the incidence of aRTill relative to competitive season 2013 was higher in 2015 (IR: 1.4; 1.0–1.9); and lower in 2016 (IR: 0.6; 0.4–0.9) and 2017 (IR: 0.5; 0.3–0.8). Travel away from home (IR: 2.2; 1.7–2.8) and travel in the last 7 days (IR: 1.9; 1.5–2.4) are associated risk markers of aRTill compared to home-based periods (p<0.0001, respectively). In the multiple regression analysis, competitive season years and travel away from home are risk markers associated with aRTill (p<0.0001). Maximum WBGT in the last 7 days (adjusted for season and travel) is not a risk marker associated with aRTill. Age, BMI, player position, illness previous year, team involved, phase of season year and number of matches played are not significant risk markers associated with aRTill in Super Rugby players.

## Conclusion

Different competitive season years and travel home/away are risk markers associated with aRTill in rugby players during the Super Rugby tournaments. These findings are important in designing and implementing of aRTill prevention strategies in rugby players.

# Abstract: O30: EXERCISE ASSOCIATED MUSCLE CRAMPING IS MORE SEVERE FOR 56KM RUNNERS COMPARED TO 21.1KM RUNNERS

**Published:** 2022-01-01

**Authors:** Esme Jordaan, Martin Schwellnus, Nicola Sewry

**Affiliations:** 1 Biostatistics Unit, South African Medical Research Council; 2 Sport, Exercise Medicine and Lifestyle Institute (SEMLI) and Department of Physiology, Faculty of Health Sciences, University of Pretoria, South Africa; 3 International Olympic Committee (IOC) Research Centre, South Africa; 4 Emeritus Professor, Faculty of Health Sciences, University of Cape Town; 5 Sport, Exercise Medicine and Lifestyle Institute (SEMLI) and Department of Physiology, Faculty of Health Sciences, University of Pretoria, South Africa

## Background

Exercise Associated Muscle Cramping (EAMC) is defined as a ‘painful spasmodic involuntary contraction of skeletal muscle that occurs during or immediately after muscular exercise’. Our previous work from the 2012–2015 Two Oceans races showed that cramping is more prevalent among 56km runners compared to 21.1km runners (56km 19% vs 21.1km 9%). The aim was to compare the severity of cramping for runners in the longer distance versus the shorter distance.

## Methodology

This was an observational study with a cross-sectional analysis. The data were from the 2012–2015 Two Oceans races (21.1km and 56km). The online pre-race medical history screening tool included a specific question related to the severity of EAMC during or immediately after running in training or competition: “If you cramp, how severe is the cramping?”. The options for the responses were “mild (15min, stop exercising)”. The 76 609 race entrants were grouped as follows into 3 race distances: (1) only 21.1km, (2) both 21.1km and 56km (21.1/56km), and (3) only 56km races over the 4 years.

## Results

The 3 race distance groups had 44 438 (58%) for 21.1km, 5 231 (6.8%) for 21.1/56km and 26 940 (35.2%) for 56km runners respectively in each group. Overall, the responses to the severity of cramping were 0.7% severe, 4.2% moderate, and 8.9% mild. The %s of severe cramping in the 3 groups were 21.1km 0.5%, 21.1/56km 0.8% and 56km 1.2%. The relative difference between the groups was as follows; The odds of 56km runners cramping being more severe is almost 3 times the odds of 21km being more severe. The odds of 21.1/56km runners cramping being more severe is 2 times the odds of a 21.1km runner cramping being more severe. The odds ratios were different for age categories but not for genders.

## Conclusion

There is a substantial difference in the severity of cramping for runners between the shorter and the longer races. This might not be true to the same extent for all age categories, and this will be investigated further.

# Abstract: O31: SPECIFIC CHRONIC ALLERGIES AS PREDICTORS OF ANNUAL MULTIPLE INJURIES IN INDIVIDUAL DISTANCE RUNNERS

**Published:** 2022-01-01

**Authors:** Sonja Swanevelder, Nicola Sewry, Martin Schwellnus, Esme Jordaan

**Affiliations:** 1 South African Medical Research Council; 2 SEMLI - University of Pretoria; 3 South African Medical Research Council

## Background

A recent publication by the authors reported that runners who have been running recreationally for >20 years and those with multiple chronic diseases or a history of allergies were at a higher risk of multiple running-related injuries (MIR). The aim was to determine if specific allergies were risk factors predictive of individual runners with a high annual MIR.

## Methodology

A retrospective, cross-sectional study at 4 annual (2012–2015) Two Oceans 21.1 km and 56km races in South Africa with 75 401 consenting race entrants. Running-related injury data were collected retrospectively through an online pre-race medical screening questionnaire. The average number of injuries for each runner by year was calculated by taking a runner’s race entry history and injury history into account and categorizing entrants into 4 MIR categories (high, intermediate, low, and very low (reference)). Follow-up multiple logistic regression modelling (odds ratios) was used to determine the role of specific allergies (any allergy, plant material allergy, animal material allergy, allergy to any type of medication) as risk factors predictive of a high MIR (average >1 injury/year).

## Results

Of all entrants, 9.2% reported at least 1 injury, and 0.4% of entrants were in the high MIR category; the incidence rate was 2.5 injuries per 10 runner-years (95% confidence interval (95%CI): 2.4–2.7). Overall, having any chronic allergy was a significant predictor of runners in the high MIR category: OR=4.5 (95%CI: 3.3–6.0; p<0.0001). Significant specific chronic allergy types predictive of runners in the high MIR category were: plant material: OR=3.5 (95%CI: 2.3–5.3; p<0.0001); animal material: OR=3.1 (95%CI: 2.0–4.9; p<0.0001); and a medication allergy OR=2.8 (95%CI: 1.8–4.3; p<0.0001), adjusting for sex, age group, race distance and taking allergy medication.

## Conclusion

Runners with a chronic allergy were at a higher risk of annually reporting multiple running-related injuries, more specifically plant material allergies, animal material allergies and an allergy to any type of medication. Further investigation should include the possible role that specifically allergy medication plays with respect to a high MIR.

# Abstract: O32: EPIDEMIOLOGY OF INJURIES IN WHEELCHAIR RUGBY AT THE LONDON 2012 AND RIO 2016 PARALYMPIC GAMES

**Published:** 2022-01-01

**Authors:** Maaike Eken, Phoebe Runciman, Wayne Hough, Wayne Derman

**Affiliations:** 1 Institute of Sport and Exercise Medicine (ISEM), Department of Sport Science, Faculty of Medicine and Health Sciences, Stellenbosch University, Cape Town, South Africa; 2 International Olympic Committee Research Centre, South Africa

## Background

Based on the nature of the sport, the risk of injuries is high in wheelchair rugby, given the sport’s high impact collisions, falls, and high risk of concussion. Previous research has alluded to high injury rates for wheelchair rugby compared to other sports at the Paralympic Games. Therefore, it is important to examine the incidence and nature of injuries in this sport, which may facilitate the development of strategies to prevent injuries. The aim of this study was to investigate the injury incidence rate and nature of injuries in wheelchair rugby during the London 2012 and Rio 2016 Paralympic Games.

## Methodology

Injuries were recorded by all medical team staff members during the London 2012 and Rio 2016 Paralympic Games, using the Web-based Injury and Illness Surveillance System (WEB-IISS) tool. The incidence rate (IR) of injuries was calculated per 1000 athlete days.

## Results

In total, 175 athletes participated in wheelchair rugby in London 2012 and Rio 2016 combined. In 29 (16.6%) of these athletes, one or more injuries were reported. A total of 38 injuries were documented, resulting in an IR of 15.5 (95%CI 25.9 – 50.1) injuries. Most athletes who reported injuries had a spinal cord injury or spina bifida (82%). Half of the injuries were acute injuries (48%), of which mostly upper limb injuries (76%). At Rio 2016, only one out of twenty reported injuries led to a time loss of two days.

## Conclusion

The results of this study showed that most of the injuries reported among wheelchair rugby athletes during the London 2012 and Rio 2016 Paralympic Games were acute upper limb injuries. Of the injuries sustained during the Rio 2016 Paralympic Games, less than 5% resulted in time lost from training and competition, most of which were hand and wrist laceration, abrasion, soft tissue bruising or haematoma. Hence, it can be suggested that wheelchair rugby resulted in a high incidence of non-time loss injuries. Future research is necessary to investigate whether prevention strategies for upper limb injuries or rule changes can lead to a reduction of injuries among wheelchair rugby athletes.

# Abstract: O33: AN EPIDEMIOLOGICAL STUDY INTO THE PREVALENCE AND TYPES OF INJURIES AFFECTING ADOLESCENT TENNIS PLAYERS IN SOUTH AFRICA

**Published:** 2022-01-01

**Authors:** Kirsty Elliott, Paola Silvia Wood, Ernst Pieter Krüger

**Affiliations:** 1 Sport, Exercise Medicine and Lifestyle Institute (SEMLI), Sport Science unit, Faculty of Health Sciences, University of Pretoria, South Africa; 2 Division of Biokinetics and Sports Science, Department of Physiology, Faculty of Health Sciences, University of Pretoria, South Africa; 3 Institute for Sports Research, University of Pretoria, South Africa

## Background

Injuries in tennis are reported as a major concern in youth tennis players worldwide, this trend has not been studied in South Africa. The aim of this study of adolescent tennis players was to determine the injury prevalence, site and types of injuries that occur and identify possible risk factors for injury in junior tennis players in South Africa.

## Methodology

A prospective epidemiological study design was used to conduct the applied research. Male and female (67%) players (n=30) aged between 13–19yrs with an average of 7.1± 1.86yrs playing experience participated. Questionnaires were used to collect: playing and injury history, daily training diaries and self-reported injury occurrence (OSICS-10 classification system) over 26 weeks. Overall Injury rate/1000 hrs of play, injured body segment, tissue type and mechanism were captured during the major competition period on the South African tennis calendar.

## Results

An injury rate of 0.357/1000 hours of play was reported with 27 new conditions and 40 recurring conditions being reported over the 26 weeks. Lower limb and trunk injuries were the most common (29.6% each), followed by upper limb injuries (22.2%) and there were no injuries to the head and neck. Muscle and tendon sprains were the most common (46%), followed by joint and ligament injuries (23%). The mechanism of the injuries reported was split between acute (55%) and chronic overuse (45%) injuries but chronic injuries accounted for (61%) of the rest days taken due to injury. The only risk factor identified to be associated with total number of injuries was the number of resting days (p=0.28).

## Conclusion

Overall injury rate recorded falls on the lower side when compared to rates found in previous studies. The trend of lower body injuries being more common than upper body occurrence was consistent with previous research. The epidemiology of injuries provides insight for the development and implementation of injury prevention programmes. Descriptive training results such as the mean training time (hrs/week), strength and conditioning training (hrs/week) and training to competition ratios should be further investigated to fully understand the major risk factors for injury occurrence.

# Abstract: O34: IMPACT OF SARS-COV2 CONTAINMENT MEASURES IN PHYSICAL ACTIVITY AND NUTRITION HABITS IN AFRICAN URBAN CENTERS: A STUDY IN MOZAMBIQUE

**Published:** 2022-01-01

**Authors:** António Prista

**Affiliations:** 1 Physical Activity and Health Research Group; 2 FEFD; 3 Universidade Pedagógica de Maputo

## Background

In response to the SARS-COV2 pandemic, a large set of measures was adopted by the authorities. These measures are expected to have a significant impact on several socio-economic and cultural habits, which include physical activity and nutrition. The assessment of this impact in opposition to its advantages in the control of the pandemic must be evaluated. Thus, the aim of this study was (1) to assess the impact of SARS-COV2 containment measures on physical activity and nutritional habits in an urban population of Mozambique and (2) to evaluate the advantages of those measures for the pandemic control.

## Methodology

A total of 7,415 men and women aged between 18 and 65 years were surveyed in 9 provincial capital cities of Mozambique by specifically trained observers about the impact of the measures on physical activity and nutritional habits. In turn, an evaluation of the impact of the measures in the evolution of the pandemic was performed by using the different official measures opposing to the subsequent evolution of rates of positive cases, hospitalization and deaths.

## Results

Almost all participants reported leaving home on a daily basis but only 5.5% for physical exercise. Almost half of the participants (48.6%) reported having been affected by their family income. Physical activity, when compared to what was habitual before the pandemic, decreased for 72.2% of people, while 27% of respondents said they eat less fruit and 29% less vegetables. In turn, the trend for positive cases, hospitalization and death rates did not show any association with the official measures.

## Conclusion

It was concluded that in the main urban centres in Mozambique, physical activity and nutritional habits were affected by the measures, although those measures did not show an association with an effective pandemic control.

# Abstract: O35: THE INCIDENCE OF ILLNESS AND INJURY IN MASTER PARALYMPIC ATHLETES: A DESCRIPTIVE COHORT STUDY OF THE >35 YEAR AGE CATEGORY IN THE 2012 TO 2018 SUMMER AND WINTER PARALYMPIC GAMES

**Published:** 2022-01-01

**Authors:** Phoebe Runciman, Melissa Janse van Vuuren, Pieter Boer, Esme Jordaan, Wayne Derman

**Affiliations:** 1 Institute of Sport and Exercise Medicine, Department of Sport Science, Stellenbosch University; 2 Cape Peninsula University of Technology, Cape Town, South Africa; 3 Biostatistics Unit, Medical Research Council, Cape Town, South Africa; 4 International Olympic Committee Research Centre ( South Africa)

## Background

There are limited studies regarding the illness and injury patterns of older Para athletes. The aim of this study was to describe illness and injury incidence in Master Paralympic athletes during the London 2012, Sochi 2014 Winter, Rio 2016, and Pyeongchang 2018 Winter Paralympic Games.

## Methodology

This study forms part of a larger epidemiological study and made use of illness and injury data regarding Master Paralympic athletes collected during the pre-competition and competition periods during the 2012–2018 Paralympic Games.

## Results

A total of 457 and 83 illnesses were reported during the Summer Games (SG) and Winter Games (WG), respectively. Some differences in illness incidence could be seen between SG and WG (London: IR 14.4; Sochi: IR 22.6; Rio: IR 11.8; Pyeongchang: IR 15.4) and illnesses in the respiratory system (IR 3.2 – 6.6), dermatologic system (2.1 – 3.8) and gastrointestinal system (1.8 – 3.1) were most common. A total of 397 and 115 injuries were reported during the SG and WG respectively. Injury IR during the WG (Sochi: IR 31.5; Pyeongchang: IR 21.1) was double compared to the SG (London: IR 12.1; Rio: IR 10.6). Upper limb (London: IR 5.9; Sochi: IR 14.1; Rio: IR 4.2; Pyeongchang: IR 11.0) and sudden onset (London: IR 5.9; Sochi: 18.8; Rio: IR 4.5; Pyeongchang: IR 16.7) injuries were two-fold higher during the WG compared with the SG.

## Conclusion

Except for Sochi 2014 WG, the incidence of illness between SG and WG were similar. The WG had a higher incidence of injuries, indicating that Master Paralympic athletes are potentially more likely to report injuries. Respiratory, dermatologic and gastrointestinal illnesses, as well as upper limb and sudden onset injuries, were prevalent in both the SG and WG.

# Abstract: O36: CLASSIFICATION OF JUNIOR ETHIOPIAN FOOTBALL PLAYERS USING ANTHROPOMETRIC AND PHYSICAL FITNESS ATTRIBUTES: DEVELOPING A PREDICTIVE MODEL

**Published:** 2022-01-01

**Authors:** Leyekun Gebru, Johan van Heerden, Orthodox Tefera

**Affiliations:** 1 Department of Biokinetics, Exercise and Leisure Sciences (Sports Science) School of Health Science, University of Kwazulu-Natal, Durban, Republic of South Africa; 2 School Academic Leader: Research School of Health Sciences Department of Biokinetics, Exercise and Leisure Sciences (Sports Science) School of Health Science, University of Kwazulu-Natal, Durban, Republic of South Africa; 3 Graduate School of Business and Leadership University of Kwazulu-Natal, Durban, Republic of South Africa

## Background

The recruitment and early selection of players into a specialised football development programme and centres of excellence have been considered as an essential factor for the long-term development of a footballing career (le Gall, Carling, Williams, & Reilly, 2010). The aim of the present study was to develop a predictive model that identifies the anthropometric and physical fitness that best predicts the status and playing positions (N=400; 15–17 years) in the Ethiopian male youth soccer development programme.

## Methodology

Anthropometric measurement in terms of mass, height, relative body fat, and derived body composition was conducted using the International Society for the Advancement of Kinanthropometry (ISAK) guidelines. Fitness was measured by sprint tests (10 meters, 20 meters and 40 meters flat), the Illinois agility test, vertical jump test and estimated VO2 max. Factorial ANOVA was used to test the relationships between players’ residence, position, and respective interaction terms. Additionally, discriminant analysis was used to identify the variables that contributed to the selections.

## Results

Sports camp residence players were significantly different in all anthropometric attributes as well as physical fitness attributes at p<0.05 except VO2 max. The percentage of players who were correctly classified in the original groups was slightly lower than the percentage calculated after the analysis was performed for the total sample (86%) and after calculation by position (86 – 90%).

## Conclusion

The study concluded that speed assessed in10m, 40m, vertical jump, and height were the discriminate attributes of players by the residential unit in Ethiopian junior football players. It is recommended that anthropometrical and physical fitness attributes are important in the assessment and selection of young football players with the potentials for higher-level performance selection.

# Abstract: O37: INJURY PROFILES DIFFER BY ATHLETE IMPAIRMENT TYPE DURING THE 2012 AND 2016 SUMMER PARALYMPIC GAMES: A COMBINED ANALYSIS OF 101 108 ATHLETE-DAYS

**Published:** 2022-01-01

**Authors:** Faatima Adam, Wayne Derman, Martin Schwellnus, Pieter Boer, Esme Jordaan, Phoebe Runciman

**Affiliations:** 1 Department of Sport Science, Faculty of Medicine and Health Sciences, Stellenbosch University (Cape Town, South Africa); 2 Institute of Sport and Exercise Medicine (ISEM), Department of Sport Science, Faculty of Medicine and Health Sciences, Stellenbosch University, Cape Town, South Africa; 3 International Olympic Committee (IOC) Research Centre ( South Africa); 4 Exercise Medicine and Lifestyle Institute (SEMLI), Faculty of Health Sciences, University of Pretoria (Pretoria, South Africa); 5 Department of Human Movement Science, Cape Peninsula University of Technology (Cape Town, South Africa); 6 Biostatistics unit, Medical Research Council, Cape Town, South Africa

## Background

Musculoskeletal injury rates in Para athletes have been shown to differ by age, sex, sport, environment, chronicity, and anatomical area. The relationship between an athletes’ medical impairment and injury rates have not yet been comprehensively studied.

## Objectives

To describe the effect of an athletes’ underlying medical impairment on their incidence of injury by age, sex, Games period, sport, chronicity, and anatomical area.

## Methodology

A combined analysis of injuries sustained by athletes during the London 2012 and Rio 2016 Summer Paralympic Games (S-PGs) was performed. A total of 7222 athletes were monitored over the two Games periods, comprising 101 108 athlete-days. Injury data were reported by impairment type: brain disorders (BD), limb deficiency (LD), neuromuscular disorders (NMD), spinal cord injuries (SCI), visual impairment (VI), and ‘all others’ (OTH: impaired passive range of movement (IPROM), intellectual impairment (II), leg length difference (LLD), short stature (SS), and unknown) impairments.

## Results

A total of 1143 injuries were reported in 980 injured athletes over both S-PGs. Athletes with VI (IR 13.6) and NMD (13.3) had high injury incidence, followed by SCI (11.1), LD (10.9) and BD (9.1). Precompetition period incidence was higher (IR 13.8) than the competition. Acute sudden onset injuries (IR 5.8) were more common than gradual onset injuries (IR 3.7), except for athletes with NMD (IR 5.9). Athletes with NMD and SCI were associated with higher rates of upper limb injuries and athletes with VI,BD and LD with lower limb injuries. The incidence (I) of injuries for the various Sports disciplines differed significantly for VI athletes (adjusted for sex and age) (p<0.0001).

## Conclusion

The findings from this study contribute toward advancing the understanding of impairment-related factors associated with injuries in athletes during summer Paralympic sports. Clinicians working with Para athletes can gain impairment-specific information for training and injury prevention. Clinicians should work around; (1) decreasing precompetition injuries in athletes with musculoskeletal impairments, (2) prioritise postural control and balance in athletes with VI, and (3) consider biomechanical compensations and assistive device usage as contributors to injury in athletes with NMD.

# Abstract: O38: MUSCLE ACTIVATION DURING THE MODIFIED CLINICAL TEST FOR SENSORY INTEGRATION AND BALANCE (MCTSIB) IN ACTIVE UNILATERAL TRANSTIBIAL AMPUTEES

**Published:** 2022-01-01

**Authors:** Sarah Arnold, Lara Grobler, Laura-Ann Furlong, Ranel Venter

**Affiliations:** 1 Stellenbosch University; 2 Limerick University

## Background

Balance tasks pose a specific challenge to UTTA after amputation often resulting in a fear of falling. There is limited understanding of postural control and muscle activation during balance tasks. Understanding the muscle activation during functional tasks could inform the evidence-based rehabilitation exercise selection. The aim of the study was to determine the muscle activation in unilateral transtibial amputees (UTTA) during the modified clinical test for sensory integration and balance.

## Methodology

Surface electromyography (Noraxon, USA) was used to determine lower limb muscle activation in six unilateral transtibial amputees (34 ± 5 years, 5 men & 1 woman) during the mCTSIB. Raw data were filtered using a high pass, low pass and bandpass eighth-order Butterworth filters. Full-wave rectification and smoothing (50ms window were applied. Peak amplitudes were determined per muscle, per activity and recordings were then normalized to the peak amplitudes. Ethical clearance and participant consent were attained prior to testing. Effect sizes (Cohen’s *d*) were used to determine meaningful practical differences.

## Results

Significant differences were found in muscle activations between surface conditions as well as between the affected and unaffected sides of the UTTA. Affected side Vastus lateralis obliques (VLO) muscle activation was greater (*d*=0.5, medium) during the eyes open on a firm surface than on a foam surface. VLO had greater activation on the affected side than the unaffected side while eyes were closed on a firm surface (*d*=0.8, Large). Affected side Bicep femoris (BF) muscle activation was greater (*d*=0.7, medium) during the eyes open on a firm surface than on a foam surface. During the Eyes open on a firm surface, the BF muscle activation was greater (*d*=0.7, medium) compared to the eyes closed on a foam surface. Tibialis anterior muscle activation was greater (*d*=0.8, large) during the eyes open on a firm surface compared to eyes closed.

## Conclusion

The importance of proprioceptive and balance training is emphasized. Evidence-based exercise selection informed by muscle activation under specific conditions has been shown to be valuable. This can contribute to reducing the fear of falls, improving prosthetic trust and improving sensory integration in UTTA. Quality of life can be maintained as the UTTA ages.

# Abstract: O39: THE COMPARISON OF LOWER LIMB KINEMATICS ACROSS STROKE RATES AMONG MALE ROWERS DURING ERGOMETER ROWING AND THE INFLUENCE OF LOWER LIMB STRENGTH

**Published:** 2022-01-01

**Authors:** Lisa Heyneke, Andrew Green, Warrick McKinon

**Affiliations:** 2 University of Johannesburg; 2 University of Witwatersrand

## Background

Rowing is a complex sport that requires an efficient technique for optimal performance and injury avoidance. The rowing stroke is a cyclic movement pattern in a closed chain. A biomechanical evaluation of rowing kinematics enables the quality of the rowing technique to be analysed. Kinematic analysis of the lower limb is important as these muscles are the primary generators of force and power in rowing. The purpose of this study was to compare lower limb kinematics during rowing at different stroke rates (20, 26, and 30 strokes/minute) among university-level male rowers.

## Methodology

Three-dimensional kinematics of 16 university-level rowers (mean age: 21.5 years, height:184.1cm, mass: 78.6kg) were recorded at a rate of 100Hz on the stationary ergometer at three different stroke rates: 20, 26, and 30 strokes/minute. Bilateral hip, knee, and ankle angles were compared using spatial parametric mapping (SMP) across the three-stroke rates (ANOVA).

## Results

The ANOVA SPM test revealed significant differences in all joint angles across the stroke rates. Both hip angles demonstrated significant differences across the stroke rates at various periods of the stroke cycle. Both knee angles were significantly different (p<0.001) across the stroke rates for the last 90% of the stroke cycle. Both ankle angles were significantly different (p<0.001) across the stroke rates for the last 80% of the stroke cycle. The post-doc analyses revealed that the largest difference in all joint angles was between stroke rates 20 and 26. For the knee and ankle angles, the least significant differences in the stroke cycle were seen between stroke rates 26 and 30.

## Conclusion

This study found that joint angles and lower limb kinematics are significantly different across the stroke rates in ergometer rowing. It is important to understand how kinematics change during the rowing stroke as the rowing technique plays a major role in success. Kinematic changes in rowing can additionally be linked to strength components such as torque and power which may further facilitate performance enhancement and injury prevention.

# Abstract: O40: THE EFFECT OF COUNTERMOVEMENT JUMP INITIATION DETECTION METHODS ON DISCRETE PERFORMANCE VARIABLES

**Published:** 2022-01-01

**Authors:** Cassidy de França, Matthew Jordan, Helen Bayne

**Affiliations:** 1 Department of Physiology, Faculty of Health Sciences, University of Pretoria, South Africa; 2 Faculty of Kinesiology, University of Calgary, Canada

## Background

The calculation of countermovement jump (CMJ) performance metrics is based on the impulse-momentum theorem that allows vertical centre of mass velocity to be determined from vertical ground reaction force (vGRF). An important methodological consideration is the detection method for true initiation of the CMJ from a stationary start. The aim of the study was to investigate if different initiation detection methods have an effect on CMJ performance variables.

## Methodology

Ethical approval was obtained to analyse the force-time data of 21 athletes who performed the CMJ as part of routine testing. Force-time data were analysed using the Shiny Vertical Jump Analysis app ( https://github.com/mattsams89/shiny-vertical-jump). Two initiation methods were applied. Bodyweight (BW): vGRF over 1-second of quiet standing. Method 1: the point where vGRF decreased to BW-5SD; Method 2: used an algorithm to search backwards from BW-5SD to determine if the inverse (BW + 5SD) occurred within the previous 100 ms. The last point before this inverse threshold was initiation. Metrics commonly used to analyse jump performance were compared between methods using paired sample t-tests.

## Results

Statistically significant differences (p < 0.05) occurred for time to take-off (0.86 ± 0.08 s; 0.80 ± 0.07 s), unweighting phase duration (392 ± 57 ms; 337 ± 41 ms), propulsion phase duration (290 ± 25 ms; 289 ± 25 ms), net impulse (2.63 ± 0.25 Ns.kg−1; 2.60 ± 2.61 Ns.kg−1), peak power (49.5 ± 6.57 W.kg−1; 49.1 ± 6.63 W.kg−1), modified reactive strength index (0.41 ± 0.08; 0.44 ± 0.09), average rate of force development (1808 ± 421 N.s−1; 2037 ± 560 N.s−1) and jump height (calculated from impulse) (0.36 ± 0.07 m; 0.34 ± 0.07 m). No statistically significant differences were observed for all other metrics analysed (jump height from flight time, braking phase duration, peak force, and force at zero velocity).

## Conclusion

Initiation detection methods significantly affect CMJ performance variables including jump height, phase durations, and timing-related metrics, but not the peak force value and therefore are not interchangeable when determining true initiation of the CMJ. Accounting for initiation with an increased force prior to decrease is recommended to calculate accurate CMJ performance and phase variables.

# Abstract: O41: ECCENTRIC MOVEMENT STRATEGY PREDICTS STRETCH-SHORTEN CYCLE PRELOAD DURING COUNTERMOVEMENT JUMPS

**Published:** 2022-01-01

**Authors:** Darius Sangari, Helen Bayne, John Cronin

**Affiliations:** 1 Department of Physiology and Sport, Exercise Medicine and Lifestyle Institute, Faculty of Health Sciences, University of Pretoria; 2 Faculty of Health and Environmental Sciences, Sports Performance Research Institute New Zealand, Auckland University of Technology.

## Background

The coupling of eccentric and concentric muscle action is common in sporting movements and is referred to as the stretch-shorten cycle (SSC). The SSC elicits a performance-enhancing effect as the height achieved in the countermovement jump (CMJ) has been observed to be higher than from concentric-only squat jumps. Net force at the end of the CMJ eccentric phase (force at zero velocity – F0V) is positive, whereas squat jumps performed from a static start where the vertical ground reaction force equals bodyweight. This may provide a quantitative indication of SSC preload. It is theorised that eccentric movement strategies (such as the amplitude and rate at which the eccentric contraction is performed) will influence the preload achieved.

## Methodology

A total of 202 competitive athletes (females: n = 83 and males: n = 119) from various sports volunteered to participate in this study. Each participant performed three CMJ’s (without arm-swing) on a dual force platform (JM6090-06, Bertec, USA) and data was collected using Forcedecks software (Vald Performance, Australia). A Pearson’s correlation was used to determine the strength of the relationship between eccentric strategy variables and relative F0V (F0V/bw). A hierarchical regression model was used to determine the predictive potential of the eccentric strategy variables. Statistical significance was set at p < 0.05. The variables selected to represent CMJ strategy were; net minimum eccentric force (Fmin), eccentric peak velocity (Vecc), relative braking impulse (Iecc/bw) and countermovement depth (CMdepth).

## Results

Large significant correlations (r > 0.50; p < 0.05) were observed between F0V/bw and Fmin, Vecc, and Iecc/bw for both male and female athletes. The hierarchical linear regression revealed that 67.1% and 63.7% of relative F0V values can be predicted using Vecc, CMdepth and Fmin for females and males, respectively.

## Conclusion

A CMJ strategy that utilizes a greater unweighting acceleration (lower Fmin and greater Vecc) and greater braking phase deceleration (greater Iecc/bw) will increase the magnitude of preload achieved (greater F0V/bw). It is suggested that practitioners aiming to improve SSC preload should consider verbal instructions and training methods that may influence eccentric movement strategies.

# Abstract: O42: CHANGE OF DIRECTION TIME VS CHANGE OF DIRECTION DEFICIT: IS THERE A NEED TO ASSESS BOTH?

**Published:** 2022-01-01

**Authors:** Christo van Zyl, Helen Bayne, Jason Tee

**Affiliations:** 1 Department of Physiology and Sport, Exercise Medicine and Lifestyle institute, Faculty Health Sciences, University of Pretoria; 2 Carnegie Applied Rugby Research (CARR) Centre, Carnegie School of Sport, Leeds Beckett University, Leeds, United Kingdom

## Background

Change of direction (COD) ability has been related to decisive moments in a team sport that could impact the outcome of a match. Change of direction is a component of agility that is described as the ability to decelerate, change movement direction, and accelerate again where no immediate reaction to a stimulus is required, thus the direction change is pre-planned. Change of direction deficit (CODD) is a calculation used to isolate the ability to change direction from the ability to sprint in a straight line. This has been suggested to provide a more isolated measure of COD ability which is not influenced by linear speed qualities. Thus, we aimed to determine the rank order relationship between COD time and CODD.

## Methodology

Forty-five athletes that consisted of male high school rugby players (n = 20), male university-level hockey players (n = 12), and female university-level hockey players (n = 13) participated in this study. Field-based testing consisted of a 20-m linear sprint (0-, 5-, 10-, and 20-m splits) and 180° (505 COD test), 90°, and 45° cutting tasks. The time between the 10-m and 20-m splits in the linear sprint test was subtracted from the time to complete the cutting task to determine the CODD. Spearman’s rank order correlation was performed between COD and CODD for the various cutting tasks.

## Results

Spearman’s rank order correlations showed nearly perfect (r = 0.94, p < 0.001), very large (r = 0.897, p < 0.001), and very large (r = 0.879, p < 0.001) relationships between COD time and CODD during the 180°, 90°, and 45° cutting tasks respectively.

## Conclusion

CODD has been suggested to isolate COD ability from sprinting ability. Our results indicate that there is a strong correlation between COD and CODD and thus the addition of CODD may not be necessary. In particular as athletes with a fast COD time will most likely have a fast CODD. However, practitioners working with athletes that have a slow COD time could find value in calculating CODD to help identify if the problem is with straight line sprinting or their ability to change direction.

# Abstract: O43: CARDIOVASCULAR AUTONOMIC NERVOUS SYSTEM-RELATED DISCRIMINATORS OF TIME TRIAL POWER OUTPUT IN ENDURANCE-TRAINED ATHLETES

**Published:** 2022-01-01

**Authors:** Ben Coetzee, Ninette Thiart, Christo Bisschoff

**Affiliations:** 1 Physical Activity, Sport and Recreation Research Focus Area, Faculty of Health Sciences, North-West University North-West University

## Background

The study aimed to determine whether the pre-test heart rate variability (HRV) and post-test heart rate recovery (HRR) parameters of endurance-trained athletes can serve as significant discriminators between higher and lower average relative time trial (TT) power outputs.

## Methodology

Ten competitive cyclists, and ten competitive middle- and long-distance male athletes participated in the study. Heart rate was measured before and after a 5-km treadmill running on a Woodway Pro XL Treadmill or a 20-km cycling TT on a Wattbike Pro Air Trainer via a Fix Polar HR Transmitter Belt and Monitor. Kubios HRV Premium software was used to analyse the series of R-R-intervals and determine the pre-test HRV. The HRR was calculated in absolute and relative values at 60 seconds post-test. The average power output of athletes during the running TT was determined by the running power equation of Van Dijk and Van Megen (2017), whereas the power output of the cycling TT was obtained from the Wattbike. The participants were allocated to a lower and higher power output group, respectively according to their TT relative power outputs. Forward stepwise discriminant analyses determined the power of different pre-test HRV- and post-test HRR-parameters to serve as discriminators of athletes and cyclists with low or high TT power outputs.

## Results

Pre-test power high frequency (HF) (%) and low frequency (LF):HF ratio were identified as the only HRV-related variables that significantly discriminated between the high- and low-power groups. The HRV-based forward stepwise discriminant analysis model was 90% accurate in classifying the participants into their respective groups. None of the HRR-related variables served as strong or significant discriminators between the low- and high-power groups.

## Conclusion

In conclusion, results suggest that Power HF (%) and LF:HF ratio can be used as significant discriminators between athletes and cyclists who can be categorized into different groups according to their average relative TT power outputs. The ANS of endurance athletes who can deliver more power during execution of the TT showed a bigger inhibition of the parasympathetic nervous system, and a simultaneous enhancement in sympathetic activity.

# Abstract: O44: PROTEIN SUPPLEMENTS IN SOUTH AFRICA: HIGH PROTEIN-CONTENT PRODUCTS OR A CASE OF MISINFORMATION?

**Published:** 2022-01-01

**Authors:** Mandisi Sithole, Gary Gabriels

**Affiliations:** 1 University of the Witwatersrand

## Background

In recent years, the popularity of protein-based supplements has increased due to increased consumer demand and claims of high-quality protein it conveys. However, studies have revealed that certain supplements may not contain the precise amounts of protein content stated on product labels. Methods such as the Dumas and Kjeldahl, which are often used to assess protein concentrations in these products, are insufficient because other nitrogen-based molecules present in the sample may interfere and result in an incorrect protein content determination. The purpose of the study was to use a multi-protein assay approach to quantify protein concentrations in protein supplement products commercially accessible in South Africa.

## Methodology

Twenty-one protein supplement products were tested in the laboratory using the BCA, Lowry, and Bradford assays

## Results

Protein concentrations (p<0.05) were lower in Bradford, BCA, and Lowry by 95%, 86 %, and 67%, respectively. Protein content determined by the Bradford assay was much lower than that determined by the other assays, owing to its limitation in identifying proteins smaller than 3000Da. There was no significant variation in protein content between the claimed values on labels and the goods tested using the Lowry assay in 33% of the cases. An interference investigation revealed that quantities of melamine, cyanuric acid, and uric acid of more than 100g.ml−1 had a minor effect on all three assays, but lower values had no effect.

## Conclusion

Based on the findings of this study, protein supplements sold in South Africa have lower concentrations of protein than what is stated on the labels. This suggests that manufacturers could be estimating protein content inaccurately or simply falsifying it.

# Abstract: O45: AGE, SKELETAL MATURITY AND GROWTH VELOCITY AS INJURY RISK FACTORS IN ELITE YOUTH FOOTBALL (SOCCER) PLAYERS AGED 11 TO 15 YEARS: A THREE-SEASON PROSPECTIVE STUDY

**Published:** 2022-01-01

**Authors:** Eirik Halvorsen Wik, Karim Chamari, Montassar Tabben, Valter Di Salvo, Warren Gregson, Roald Bahr

**Affiliations:** 1 Institute of Sport and Exercise Medicine, Faculty of Medicine and Health Sciences, Stellenbosch University, Tygerberg, South Africa; 2 Aspetar Sports Injury and Illness Prevention Programme, Aspetar Orthopaedic and Sports Medicine Hospital, Doha, Qatar; 3 Football Performance and Science Department, Aspire Academy, Doha, Qatar; 4 Oslo Sports Trauma Research Center, Department of Sports Medicine, Norwegian School of Sport Sciences, Oslo, Norway

## Background

Growth and maturation are considered risk factors for injury in youth football, yet firm conclusions cannot be drawn from the existing research. Our aim was to explore the main and combined effects of age, skeletal maturity and growth velocity on injury risk in elite academy players.

## Methodology

Anthropometric (triannual height and body mass assessments), maturity (Fels skeletal age from annual hand/wrist x-rays), injury (time-loss injuries recorded by team physiotherapists) and exposure (individual training/match minutes recorded by team sport scientists) data were collected prospectively over three seasons in an elite academy, including 95 male players (11–15 years). We compared the relative model quality of mixed-effects logistic regression models, with growth velocity (cm/year or kg/year) for 223 growth intervals (Start to Mid-season or Mid-season to End, average 113 days) included as fixed effects, and adjusted for age or maturity plus load (average hours/week). Associations were interpreted as practically relevant based on the confidence interval for odds ratios, using thresholds of 0.90 and 1.11 to define small beneficial and harmful effects, respectively.

## Results

During the growth intervals, 161 index injuries and 21712 exposure hours were recorded. Change in body mass combined with maturity best explained overall and gradual onset injury risk, while change in height combined with maturity best explained the risk of sudden onset, bone tissue and physis injuries. No growth × age/maturity interaction effects were seen. Older age was associated with small harmful effects on overall (OR adjusted for stature change: 2.61, 95%CI: 1.15–5.91) and sudden onset (OR adjusted for body mass change: 1.98, 1.17–3.37) injury risk. Significant associations (p<0.05) were observed for body mass and maturity on sudden onset injuries, and for load on gradual onset, bone tissue and physis injuries; however, these were not considered practically relevant based on our predefined thresholds.

## Conclusion

Our findings could not support changes in height or body mass over an academy semester as injury risk factors. Older age was, however, associated with an increased risk of overall and sudden onset injuries. Future studies should strive to include larger samples, starting from pre-adolescence, to enable within-subject analyses and better understand the relationship between growth, maturation and injuries.

# Abstract: O46: DEVELOPMENT OF A TOOLKIT TO ASSESS HEALTH-RELATED QUALITY OF LIFE AMONGST PATIENTS WITH SELECTED NONCOMMUNICABLE DISEASES

**Published:** 2022-01-01

**Authors:** Lynn Smith, Heather Morris-Eyton

**Affiliations:** 1 University of Johannesburg

## Background

Although South Africa has made significant progress in reducing the risk associated with noncommunicable diseases (NCDs), the country still faces health inequities and inequalities within the healthcare sector. Low- and middle-income countries, such as South Africa, are called to expand their healthcare capacities to respond effectively to the increased burden of NCDs. An integrated approach to the broad spectrum of NCDs may provide the most cost-effective method for treatment, with a focus on health-related quality of life (HRQoL). This study aimed at developing a toolkit to assess the HRQoL of patients with hypertension, type 2 diabetes and cardiovascular disease.

## Methodology

A sequential and exploratory mixed method research design was utilised. Focus group discussions and semi-structured interviews were conducted with an expert panel (n=12). This process elucidated the development of a questionnaire assessing HRQoL. This questionnaire was piloted and amended according to the feedback received. It was administered to patients (n3=257) across two-time intervals, three weeks apart. Principle component analysis was performed on all items and results were used to determine validity and reliability using the Pearson and Spearman correlation coefficients, the interclass correlation coefficient (ICC) and the coefficient of repeatability (CR).

## Results

The toolkit consists of a long (37 items) and short (25 items) form questionnaire. Both questionnaires yielded an excellent Pearson’s r (0.89*; 0.89*), Spearman’s rho (0.88*; 0.89*), and ICC (0.94; 0.94). The coefficient of repeatability for both the long and short form were considered acceptable, at ±12.04% and ±12.50%, respectively.

## Conclusion

The toolkit questionnaires are highly reliable and provides healthcare practitioners with a starting point for treatment, aiding in the prescription of individualised, multifactorial treatment programmes. These are tailored specifically to improve HRQoL and the progression towards healthier lifestyles. In addition, the results of this study encourages a palliative approach to healthcare, improving the patients’ HRQoL through the prevention and relief of disease specific suffering.

# Abstract: O47: BODY COMPOSITION WITH NUCLEAR AND ISOTOPE TECHNOLOGY APPLICATION WITHIN THE AFRICAN REGION

**Published:** 2022-01-01

**Authors:** M.A. Monyeki

**Affiliations:** 1 Physical Activity, Sport and Recreation Research Focus Area, Faculty of Health Sciences, North-West University, Potchefstroom, South Africa

## Background

Human body composition measurements are methods suitable for nutritional assessment and are of interest to sports scientists, nutritionists and health professionals. The increasing prevalence of childhood obesity, inactivity and lifestyle diseases, compiles increasing need for body composition methods with greater sensitivity and precision.

## Purpose

The purpose of the study was to determine the level of agreement between body size self-perception and actual body size determined by body mass index (BMI) and body fatness measured by the deuterium dilution method (DDM); and compares body fat (%BF) determined using Bioelectrical Impedance Analysis (BIA) and %BF predicted from skinfolds equations in South African children.

## Methods

A cross-sectional study with 299 (125 boys; 174 girls) 6- to 8-year children was performed. Perceived body image was assessed silhouettes. Total body water and fat free mass were determined with Deuterium Oxide Dilution (DDM), and predicted using Bodystat (1500) with 50kHz frequency. Bland Altman plots determined the level of agreements. Statistical Package for Social Sciences (SPSS V_27_^®^) analysed the data.

## Results

BMI z-scores, thinness grade 1 (12%), thinness grade 2 (3%), overweight (9%), obese (2%); body image, underweight (32%), overweight (9%), and obese (2%) perceived their body size as underweight, normal, overweight, and obese; and DDM, 2.5% underweight, overweight (22%), obese 30%. The application of silhouettes and BMI resulted in either overestimation or underestimation of own body size. Level of agreement between body size perception, body fatness, and BMI was poor. Significant paired mean differences were found for body mass index (BIA) and Slaughter (t_201_ =33.896, p<0.001), Wickramasinghe (t_201_ = 4.217, p<0.001), and Dezenberg (t_201_ =19.910, p<0.001). The Blant-Altman plots show relatively large positive and negative deviations from the mean difference lines, and trends of systematic under-and over-estimation of %BF across the %BF spectrum.

## Conclusion

Perceived body image and BMI (which remains a practical tool for obesity surveillance, it should be consider in future studies) over or underestimated body fatness, whilst the criterion methods provided a good body fatness classification. Scalable measures to allow for more accurate self-assessment are urgently needed–one approach is behavior change communication at all levels. As such, age-specific %BF equation incorporating optimum methods using DDM are needed.

# Abstract: O48: PHYSICAL FITNESS OF SCHOOL-AGE CHILDREN AND YOUTH LIVING IN AND ISLAND IN MOZAMBIQUE

**Published:** 2022-01-01

**Authors:** Gomes Nhaca, Timoteo Daca, Antonio Prista

**Affiliations:** 1 Physical Activity and Health Research Group, Faculty of Physical Education and Sports, Pedagogical University of Maputo, Mozambique

## Background

Physical fitness (PF) is associated to health and performance being important from the early days of life. The pattern of PF in school age fitness in an Island environment in Mozambique are unknown.

### AIM

To evaluate the levels of Physical Fitness of school-age children and young living in an Island environment

## Methodology

The study is part of the Human Biological Variability project in Mozambique and was carried out in “Inhaca” Island in 2019. Sample was composed by 740 children and young aged 6–17 (Boys=378; Girls=362). The standardized protocols of the AAHPERD (mile run and sit and reach) and FITNESSGRAM (horizontal impulsion, manual dynamometry, abdominal resistance strength and 10×5 meters sprint) batteries were applied. ANCOVA was used to analyzed data as a function of sex having age as covariate. Comparison with other Mozambican studies in rural and urban areas were made using One-Way ANOVA

## Results

Age-adjusted performance indicated significant differences between boys and girls in hand dynamometry (p=0.001), horizontal jump (p=0.001), 10×5 meter sprint (p=0.014), mile run (p=0.001), with the boys presenting better performance in all tests with exception for flexibility (p=0.001). In both boys and girls, the “Inhaca” Island sample outperformed its mainland peers in the abdominal resistance strength and handgrip strength tests (p=0.001). In the one mile run test the participants from “Inhaca” Island performed identical to the rural area but better than the urban area (p=0.001).

## Conclusion

School aged children living in the “Inhaca” Island have a good fitness performance relative to urban peers suggesting an important impact effect of the environment.

# Abstract: O49: ASSOCIATIONS BETWEEN BODY COMPOSITION, PHYSICAL ACTIVITY, INTIMA-MEDIA THICKNESS, AND CARDIO-METABOLIC RISK FACTORS IN A COHORT OF TEACHERS: THE SABPA-STUDY

**Published:** 2022-01-01

**Authors:** Tamrin Veldsman, Mariette Swanepoel, Johanna S Brits, Makama A Monyeki

**Affiliations:** 1 Physical Activity, Sport and Recreation Research Focus Area (PhASRec), Faculty of Health Sciences, North-West University, Potchefstroom, South Africa; 2 Hypertension in Africa Research Team (HART), North-West University, Potchefstroom, South Africa

## Background

Obesity and physical inactivity are linked with high prevalence of non-communicable diseases (NCDs) of lifestyle, and NCDs are a significant public health concerns in adults; in the 21st century. The study therefore investigated the associations between body composition, physical activity (PA), cardio-metabolic risk factors and carotid intima-media thickness (CIMT) in teachers.

## Methodology

A cross-sectional study design; on a data of 216 teachers (104 males; 112 females) from the Sympathetic Activity and Ambulatory Blood Pressure in Africans (SABPA) prospective cohort study from the Dr Kenneth Kaunda District, North West Province of South Africa was used. Body mass index (BMI), waist circumference (WC), waist-to-height ratio (WtHR) as measures of body composition, and completed 7-day ActiHeart PA data according to standard procedures were measured. SonoSite Micromaxx ultrasound measured CIMT. The Joint Interim Statement classifications were applied for Metabolic syndrome. The Statistical Package for Social Sciences (SPSS) version 27 analysed the data.

## Results

Two thirds of the participants respectively were sedentary and participated in light-intensity PA. Twenty-nine percent per cent of the teachers had cardio-metabolic syndrome, and male teachers significantly (p<0.05) had a high mean value for CITM. WC positively and significantly (β = 0.151, p = 0.027) related to CIMT. In the total sample, a weak significant positive relationship between WC and triglycerides (r = 0.16; p = 0.02) was observed. CIMT inversely associated with total energy expenditure (r = −0.31; p = 0.05) in sedentary male teachers. Additionally, PA negatively associated with triglycerides (r = −0.29; p = 0.02), gamma-glutamyl transferase (GGT) (r=−0.25; p=0.06). After adjustments for age group, self-reported smoking and alcohol use, a weak significant negative relationship between mean 7-day awake METs and triglycerides (r = −0.28; p < 0.01) remained

## Conclusion

CIMT positively associated with WC. Participation in light PA associated with lower CIMT, triglycerides and GGT. The high cardiovascular risk profile diminished the protective role of PA. Physical activity intervention studies are recommended to determine effective interventions to provide information on how to limit the development of atherosclerosis.

# Abstract: O50: PHYSIOTHERAPISTS’ EXPERIENCES OF RUGBY-RELATED CONCUSSION MANAGEMENT IN THE COMMUNITY

**Published:** 2022-01-01

**Authors:** Marelise Badenhorst, Danielle M. Salmon, Gisela Sole, Simon Walters, Johna Register-Mihalik, Zachary Yukio Kerr, John Sullivan, Chris Whatman

**Affiliations:** 1 Sports Performance Research Institute New Zealand, School of Sport and Recreation, Auckland University of Technology, New Zealand; 2 Injury Prevention and Player Welfare, New Zealand Rugby, Wellington, New Zealand; 3 Centre of Health, Activity, Rehabilitation Research, School of Physiotherapy, University of Otago; 4 Department of Exercise and Sport Science, University of North Carolina at Chapel Hill, Chapel Hill, USA

## Background

New Zealand Rugby implemented a concussion management pathway to improve concussion management at the community level. Physiotherapists often play a key role in the identification, immediate- and long-term management of concussions. The aim of this study was to explore physiotherapists’ experiences of rugby-related concussion management, as part of the concussion management pathway, to further inform New Zealand Rugby’s concussion strategies and subsequently aid future adoption and sustainability.

## Methodology

We adopted a pragmatic, descriptive qualitative approach to explore the perceptions of physiotherapists involved in the pathway. At the end of the 2019 rugby season, semi-structured interviews and focus groups were conducted with twenty-four physiotherapists in three geographically and socioeconomically diverse regions in New Zealand (NZ). Thematic analysis was used to analyse data.

## Results

Four themes were identified that play a role in either facilitating optimal concussion management or causing high levels of pressure perceived by physiotherapists. These were: i) ‘walking the tight rope between player welfare and performance’ represented participants’ perceptions of the balancing act between different attitudes and priorities of the various rugby stakeholders (whether they prioritised team performance, or player welfare); ii) physiotherapists’ perceptions of their own authority within the team and their concussion management responsibilities; iii) the importance of multi-directional communication in the management of concussion, and iv) the influence of context, which included the complexity of concussion injuries, knowledge of the physiotherapist and team, work-load and resources to support the physiotherapist and ease of access to a medical doctor.

## Conclusion

Physiotherapists had positive attitudes toward the concussion management pathway and are well-positioned to play an essential role in this respect. However, strategies are needed to align all stakeholders’ attitudes around player welfare and address persisting challenges. Such strategies should also consider experienced physiotherapists mentoring young or inexperienced physiotherapists to improve self-confidence for taking over team roles. The findings of this study also support the value of clear protocols, such as the concussion management pathway, in supporting physiotherapists to deliver effective concussion care. For the pathway’s future sustainability, additional support may be needed to enhance communication between stakeholders, thereby saving valuable time for the physiotherapist and, generally, facilitating the delivery of their duties.

# Abstract: O51: HEADING EXPOSURE IN PROFESSIONAL FOOTBALL TRAINING

**Published:** 2022-01-01

**Authors:** Steve den Hollander, Gino Kerkhoffs, Vincent Gouttebarge

**Affiliations:** 1 FIFPRO; 2 Amsterdam UMC, University of Amsterdam; 3 ACHSS, AMC/VUmc IOC Research Centre; 4 Section Sports Medicine, UP

## Background

Heading the ball has been identified as a risk factor for concussion in association football. However, the exposure to heading, and the g-force of the impacts, are understudied. While ranging from 3.6 (midfielders in Ligue 1) to 9.2 (defenders in English Championship) per player per match, the number of headers a player makes during training remains unknown. Furthermore, self-reported exposure to heading has been shown to be unreliable. Therefore, this study aimed to assess the exposure of players to heading in training through objective measures and determine the reliability of self-reported exposure to heading.

## Methodology

Sixteen professional footballers took part in a training camp. The camp consisted of 4 training sessions, 2 per day, followed by a match. All training and match sessions were filmed, and the number of headers each player made was noted, retrospectively, by a video analyst. For each observed header, the player who made the header, the intentionality of the header, and the timestamp, were recorded. During all training and match sessions, the players were equipped with an impact tracker, to quantify the G-Force of the headers. After each session, the players were asked to recall how many headers they executed during the session.

## Results

Players were exposed to 2.2 headers per player per training session, with an average G-force of 15G, and 2.4 headers per play in the match, with an average force of 26G. Defenders made more headers in training (n=3.3, G-force=15G) and matches (n=3.8, G-force=19G) than forwards (training: n=2.6, G-force=17G; matches: n=3.0, G-force=34G) and midfielders (training: n=2.6, G-force=20G; matches: n=3.0, G-force=29G) per player per session. 3% of headers were unintentional, with an average G-force of 70G. The reliability of the self-reported exposure to headers was good (Intra class coefficient = 0.77).

## Conclusion

Players were exposed to fewer headers in training than in the match, with lower impact forces. Although defenders made more headers in training and matches, compared to midfielders and forwards, the impact of these headers was less. Further research is required to understand the relationships between types of headers, impact forces, and mechanisms of heading related concussions.

# Abstract: O52: REPORTING BEHAVIOURS AND ATTITUDES TO CONCUSSION AMONG HIGH-SCHOOL RUGBY PLAYERS IN KWAZULU-NATAL, SOUTH AFRICA

**Published:** 2022-01-01

**Authors:** Johan van Heerden, Cameron Scullard

**Affiliations:** 1 Dpt Biokinetics, UKZN, South Africa; 2 Institute of Sports and Preventive Medicine, Saarland University, Germany

## Background

Concussion in the sport of rugby has received much attention and many interventions to improve injury identification and management at a professional and semi-professional level. However, there is limited literature into the attitudes, perceptions and motives towards concussion injury acknowledgement and reporting with the high-school rugby population. The popularity of the sport in South-African schools, along with the rapid professional progression of the game at this level, warrants further investigation into determining how players value, or disregard, their safety for the sake of the sport. The aim of the study was to determine the attitudes and perceptions, and reporting behaviours thereof, towards concussion among high-school rugby players.

## Methodology

This descriptive cross-sectional survey comprised of 667 players (average age of 15.4±1. yrs) from KwaZulu-Natal schools and utilized a modified RoCKA-ST questionnaire which allowed for the formulation of a Concussion Attitude Index (CAI) and Concussion Reporting Index (CRI).

## Results

Less than half (n= 330; 49.5%) of the players reported having previously received concussion education and only 21.4% (p≤0.0001) of their personal knowledge of concussion was rated in the safe category. The players reported a 64.77% rating on the overall CAI which indicates a relatively good regard of the risks of concussion, However, the CRI revealed a much lower safety rating of 50.4%, and a low (44.1%) score for the attitudinal aspect of the CRI, thus reflecting poor reporting behaviours. A large proportion of players (75–91% ) reported that experiencing or reporting a concussion made them feel or look weak and that did not report concussion in fear of letting the team down or missing a subsequent match (66%).

## Conclusion

Although being aware of the risks of concussions, players expressed poor self-efficacy in experiencing and reporting concussions, leading to correspondingly poor concussion reporting practices. The majority of the players thus value participation and the risk of losing their place in the team, above their own safety. This ill-conceived loyalty of non-disclosure of concussion being seen as a “badge of honour”, among players, is concerning as it places them at serious short- and long-term risk.

# Abstract: O53: INJURY AND ILLNESS RISK PROFILES IN OLDER ACTIVE INDIVIDUALS: A SYSTEMATIC REVIEW

**Published:** 2022-01-01

**Authors:** Lovemore Kunorozva, Phoebe Runciman

**Affiliations:** 1 Institute of Sport and Exercise Medicine (ISEM), Division of Orthopaedic Surgery, Faculty of Medicine and Health Sciences, Stellenbosch University, Stellenbosch, South Africa

## Background

With increased physical activity, the burden of physical activity related injuries and illnesses may increase. Yet, only a limited number of studies investigating the epidemiology of illness and injury profiles in older active individuals with disability exist.

## Methodology

A comprehensive literature search of injury and illness risk profiles in older active individuals with and without a disability was conducted employing PubMed, Ebscohost and Web of Science databases up to 31 July 2021. Each of the articles was independently reviewed for relevance and inclusionary criteria, with 11 studies meeting these criteria.

## Results

Overall, the quality of the included studies was excellent. Respiratory, skin and sub-cutaneous, and digestive system illnesses were the commonly reported illnesses. Chronic overuse injuries in the upper extremities including shoulders (32%), chest (13%) and elbow (13%) were frequently reported. Sport and/or impairment type were the most frequently reported contributor to injury.

## Conclusion

The findings of this review indicate that: 1) sport and/or impairment type are the main contributors to both injury and illness and not age or sex; although older individuals exhibited a higher prevalence of chronic overuse injuries; and 2) older individuals have a slightly higher risk of developing illnesses, with respiratory, skin and sub-cutaneous, as well as digestive system illnesses most common. There is a need for further research on injury and/or illness profiles in older individuals, as this would allow clinicians to provide better rehabilitative and pre-habilitative care. Additionally, this would provide important information on predicting illness and injury risks and help further develop preventative strategies.

# Abstract: C01: COMPETE ANDROGEN INSENSITIVITY SYNDROME: AN ONGOING COMPETITION REGULATIONS, ETHICS AND FAIRNESS DILEMMA

**Published:** 2022-01-01

**Authors:** Lone Bogwasi

**Affiliations:** Section Sports Medicine, Faculty of Health Sciences, University of Pretoria, Pretoria, South Africa, CAF Medical committee member, Botswana Football Association Medical committee member

## History

23/F football athlete identified on routine pre-competition medical screening and sex testing (gender verification).

### Background

Primary amenorrhea, hoarseness of voice, no hirsutism

Investigated for eligibility to compete in a female football continental competition

## Physical Findings

Lean built and ectomorph structure.

Weight: 45.3 kg

Height: 167cm

BMI: 16.2

Body fat: 14.8%

Muscle mass: 36.6 kg

Bone mass: 2 kg

Physique rating: 7

Hoarse voice, No hirsutism, Flat forehead, prominent cheek bone (zygomatic bones).

Small breasts (almost flat chest)

Female genitalia, large labia and clitoris slightly larger than normal.

small palpable masses on B/L inguinal area

## Differential Diagnosis/Hypothesis

Sexual Ambiguity

## Test and Results

Testosterone levels:

24/09/2021 - 43ng/L

02/2022 - 39ng/L

FSH: 84.18 mlU/mL

LH: 5.10 mlU/mL

Progesterone: 1.19ng/mL

17b oestradiol: 139.19 pmol/L

Prolactin 13.20ng/mL

Chromosomal analysis: XY genotype, no gross structural abnormalities

Ultrasound scan findings: The uterus could not be exclusively identified on sonar. No fluid collection seen in the pelvis. There are no obvious pelvic masses or cysts seen. Urinary bladder well distended with a normal outline and no masses.

MRI scan findings:

Liver, biliary system, spleen, kidneys all normal.

Normal stomach, bowel loops and rectum. No intrabdominal lymphadenopathy.

A phallus is presently attached to ischiopubic rami. This appears indeterminate between a micropenis and an unusually enlarged clitoris. The vagina cervix and uterus are not visualized.

The prostate is not visualized, uterus and ovaries not visualized. No testis is seen. No fluid in cul-de-sac.

No significant inguinal lymphadenopathy. No sizeable hernia noted.

## Final/Working Diagnosis

Complete Androgen Insensitivity Syndrome

## Treatment and Outcomes

Based on the outcome of CAIS. She was referred to an endocrinologist who suggested the testosterone level although high does not offer any athletic advantage.

The competition rules for CAF/FIFA women football do not make her eligible to participate. The regulations are for female vs female and male vs male participation.

The options for intervention include:

1. Surgery (removal of undescended testes)
2. Hormonal therapy
3. Maintain hormonal levels for a female

Current Treatment plan:

1. The sad news was broken to her and the athlete was informed about the finding and then taken through the available options and side effects. Currently, she is thinking through the options and hasn’t made a decision yet.
2. Continued psychotherapy in place
3. Gynaecologist and surgical reviews once she decides on the operative route (She was also made aware of the risk of cancer from undescended testes).
4. Endocrinologist follow-up sessions
5. Dietician

A further treatment plan will be based on her decision.

# Abstract: C02: EXERTIONAL CARDIOPULMONARY SYMPTOMS FOLLOWING VIRAL URTI’S – TAKE IT TO HEART

**Published:** 2022-01-01

**Authors:** Marcel Jooste

**Affiliations:** 1 Sport Exercise Medicine and Lifestyle Institute (SEMLI) and Section Sports Medicine, Faculty of Health Sciences, University of Pretoria

## History

A 17-year-old national level athlete, with no comorbidities, presented with complaints of persistent, exertional cardiopulmonary symptoms 7 weeks following a suspected viral acute respiratory infection (ARinf) During the acute phase on the infection, she experienced symptoms confined to the upper respiratory tract, she was treated as presumed SARS-CoV-2 infection based on symptoms without a confirmatory nasopharyngeal swab. She continued to train during the symptomatic phase of infection. One month after the ARinf she started experiencing sharp, exertional chest pain, excessive fatigue during exercise, and an elevated resting heart rate. These symptoms persisted despite resting from exercise for 3 weeks, which led her to seek further medical advice. The patient denied any significant psychosocial stressors during this time. She had no family history of cardiovascular disease.

## Physical Findings

Vital signs: HR 71, BP 129/80, RR 12, Sats 99%. General examination was normal. Cardiovascular examination revealed no evidence of a murmur even with dynamic movements, no pericardial rub nor any signs of oedema. All distal pulses were equal with normal rhythm, no radio-femoral delay. Respiratory examination was normal with no pain with inspiration, nor tenderness to palpation of the costochondral junctions. Otolaryngological and abdominal examinations did not reveal any significant findings.

## Differential Diagnosis/Hypothesis

Costochondritis

Viral myocarditis

Viral pericarditis

Anxiety

Pulmonary embolism

Arrythmia

## Test and Results

Bloods:

FBC = normal

CRP = 1 mg/l

ESR = 4 mm/hr

hs Troponin T = <5

TSH = 1.35 mIU/L

T4 = 11.3 pmol/L

Ferritin = 21 ng/ml

Resting ECG: HR 71 Sinus arrythmia. Widespread ST elevation involving the inferolateral leads with reciprocal PR segment depression in the inferior leads. Down-sloping T-P segment (Spodick sign) in the inferior leads.

Echocardiogram: normal

Cardiac MRI: normal heart size and function. Mild pericardial enhancement of the surface overlying the anterior as well as left ventricle free wall, suggestive of pericarditis changes. No discrete pericardial effusion or thickening could be noted.

## Final/Working Diagnosis

Subacute viral pericarditis

## Treatment and Outcomes

A stress ECG was performed to assess the physiological response to limited exercise. There was a blunted systolic blood pressure response to exercise, normal heart rate recovery, no arrythmias, no ST-segment changes with exercise. The patient was counselled on the prognosis and risks of strenuous exercise during active phase of the condition. She was started on Ibuprofen and colchicine.

Recommendation was to rest and perform only light-intensity exercise for one month until follow-up consultation. If asymptomatic at follow-up, then to perform limited stress ECG.

# Abstract: C03: VERTIGO IN A HOCKEY PLAYER

**Published:** 2022-01-01

**Authors:** Jo-Anne Kirby

**Affiliations:** 1 Stellenbosch University

## History

A 21-year-old collegiate field hockey player developed vertigo during an international tournament. At first, she felt dizzy and faint but then it became rotational vertigo which would last 1s and come back approximately every 30s during and after exercise. She had no ear pain or hearing loss but on initial evaluation had redness of the ear, otitis was suspected and she was placed on antibiotics and anti-virals. She continued to play. At another tournament, she began vomiting. In addition to vertigo, she was now unable to run in a straight line after a direction change and had difficulty seeing the ball approach. Despite this, she continued to play in a series of tournaments. She felt as though she was adapting, the vertigo was reducing but was replaced by headaches and altered hearing.

## Physical Findings

On clinical examination she had horizontal nystagmus in both eyes, normal eye movements and normal neurological and cardiovascular examinations. She had redness of the tympanum at her first examination cleared on follow-up

## Differential Diagnosis/Hypothesis

Otitis Media/Interna

Vertigo of peripheral origin e.g. BPPV

Vertigo of central origin

Concussion

## Test and Results

Brain MRI revealed a well-defined, lobulated right cerebro-pontine angle mass lesion with internal inhomogeneity with a significant mass effect on the brain stem, which is deviated to the left and a subtle mass effect and encasement of the associated cranial nerves.

## Final/Working Diagnosis

Intracranial epidermoid cyst with an incidental finding of a cystic lesion within/in close association with the posterior pituitary.

## Treatment and Outcomes

This was surgically removed leaving 10% of the lining in areas of critical structures. Six weeks later she returned to academic activities. Rehabilitation started with neck range of movement exercises followed by strengthening and running. She returned to competitive sport by the end of the season and has been asymptomatic since.

# Abstract: C04: PERICARDITIS - VIRAL ENTERITIS OR COVID19 TO BLAME?

**Published:** 2022-01-01

**Authors:** Lervasen Pillay

**Affiliations:** 1 University of Pretoria, University of Witwatersrand, University of Amsterdam

## History

The patient presented with a viral bout of diarrhoea in December 2020. COVID19 infection was excluded and treated with probiotics and anti-diarrhoea medication. There were no complications and the athlete returned to activity as soon as the symptoms settled. Upon return, palpitations on exertion (very occasional) were noted but ignored. A diagnosis of COVID19 was made 5 months later (after having symptoms for 5 days which included loss of taste, fever, sore throat and slight cough). This was treated symptomatically and settled uneventfully. However, the palpitations worsened. As per team protocol, the patient presented for a post COVID19 infection medical clearance.

## Physical Findings

Initial clinical evaluation revealed normal vitals - Pulse rate of 65/min, BP 110/72, O2 saturations 98% on room air. There was a pericardial rub. The rest ECG revealed a right bundle branch block. Due to clinical findings, blood tests were done and a referral to a sports cardiologist.

## Differential Diagnosis/Hypothesis

Pericarditis/Myocarditis

## Test and Results

U and E, CKMB, FBC, CRP, Trop-T were all normal. Cardiac echo revealed a mild pericardial effusion with no tamponade. Left ventricle ejection fraction was 56% and there were no abnormal wall movements. Contrast cardiac MRI revealed a 4 – 4.5mm pericardial effusion and post-contrast pericardial enhancement confirming inflammatory pericarditis.

## Final/Working Diagnosis

Pericarditis

## Treatment and Outcomes

The patient refrained from moderate to high-intensity exertional activity for 3 months. Medical treatment consisted of Colchicine 0.5mg daily and Ibuprofen 200mg bd for 3 months. Medication was adjusted according to any side effects experienced. A cardiologist review was done after 3 months and was given the clearance to return to sport. A progressive return to play sport-specific conditioning program was implemented and returned to full activity after 6 weeks. There have been no further complaints. In light of COVID19 and the recent highlight on cardiac inflammation, it is important for clinicians to be reminded that cardiac inflammatory conditions can also be caused by other viral infections.

# Abstract: C05: "BACK TO BASICS": SPINAL PAIN IN A RECREATIONAL RUNNER

**Published:** 2022-01-01

**Authors:** Karen Schwabe

**Affiliations:** 1 Stellenbosch University

## History

This is a case presentation of an 81-year-old recreational male runner, who was in training for an international 5 km race. He was previously well, with no known underlying diseases. He sustained an injury of the thoracic spine in the gym with weight training. Initially, he consulted his GP about this injury, who diagnosed musculoskeletal back pain. At that stage, basic bloods and an XR of the thoracic spine were done, which were all normal. He was treated with NSAIM and advised to rest. Initially, he responded to rest and the medication, but the pain recurred. Subsequently, he was referred to a physiotherapist, with variable response. After a month, the pain increased, with the patient now experiencing night pain, but no other systemic symptoms. He was booked to see a neurosurgeon at this point. Whilst waiting for the appointment, he developed intractable back pain and weakness and numbness of the lower limbs - as an acute episode. At this point, he presented to the emergency department.

## Physical Findings

Initially: Central back pain level T3. Neurologically intact

ER: level T3 motoric and sensory fall-out, progressing, with urinary retention and no rectal tonus.

## Differential Diagnosis/Hypothesis

Musculoskeletal back pain secondary to a gym injury

Thoracic disc disease

Infection

Neoplastic

## Test and Results

CXR: initial normal (Dec 2021)

FBC: initial normal (Dec 2021)

MRI: T3 vertebral collapse with cord compression (Jan 2022)

CT chest: bilateral pleural effusions, multiple pulmonary nodules bilateral, small pericardial effusion

Gastroscopy: Biopsy - adenocarcinoma

## Final/Working Diagnosis

Metastatic spinal cord (T3) disease with vertebral collapse and complete motoric and sensory fall-out at this level.

## Treatment and Outcomes

Emergency decompression at level T3 and surgical screws T2 & T4

Radiation therapy 10 sessions (colon)

Chemotherapy

Step-down rehab facility

# Abstract: C06: AN UNUSUAL CASE OF A FEMORAL BONE STRESS INJURY IN A HIGH SCHOOL FEMALE HOCKEY PLAYER

**Published:** 2022-01-01

**Authors:** Karen Schwabe

**Affiliations:** 1 Stellenbosch University

## History

A 17-year-old female hockey player, sustained an injury to the left upper leg during a hockey tournament. She fell onto the right hip and injured left upper leg/felt a ‘pull’ in the proximal-medial area of the groin. She continued to play ‘through’ the pain for the rest of the game but did not participate further in the tournament games due to the discomfort.

She consulted a sports physician 5 days later. Clinically a grade 1 left adductor brevis was diagnosed and an ultrasound confirmed a proximal adductor strain. The pelvis XR was normal. At this point she was referred for physiotherapy rehabilitation, rest and to use of 1 crutch. After 10 days there was no clinical or functional improvement and her pain in weight bearing increased.

Further re-evaluation now showed bony pain of the left proximal femur and she was referred for a MRI scan. The MRI revealed extensive bone stress injury of the proximal shaft of the left femur.

She was previously healthy with no known medical diseases and no significant previous injuries. Vegetarian

## Physical Findings

Initial consultation: grade 1 left sided adductor tear

Follow-up consultation: bony pain on the femoral shaft (with improvement on the adductor strength).

Inability to now weight-bear without pain. Antalgic gait.

## Differential Diagnosis/Hypothesis

Adductor tear

Avulsion injury

Referred pain from the hip/labral pathology

Pelvic/femoral bone stress

## Test and Results

Pelvis XR: normal

Ultrasound left groin:

MRI Pelvis: Significant bone stress injury involving the subtrochanteric proximal shaft of the left femur, Fredrickson grade 4A. Bilateral acute iliopsoas musculotendinous strain patterns. Subtle common adductor insertional tendinopathy with no evidence of associated tear.

Bloods: Parathyroid hormone:low normal, Vitamin D: low normal Ferritin, FBC, u&e, Vit B12, TSH, Mg2+, Po4−, Ca2+ all normal.

## Final/Working Diagnosis

Severe overuse bone stress injury of the proximal femur

(RED-S not suspected)

## Treatment and Outcomes

Current treatment: non-weight-bearing, Vit D supplementation, physiotherapy

Future treatment plan: 3 months of partial weight-bearing, then gradual post loading and stretching.

After 3 months of gradual functional strength training and field work depending on progress.

Recheck the PTH and Vit D levels at 3 months

Dietician referral as the patient is a vegetarian

*Clinical response yet to be determined
